# Meta genetic analysis of melon sweetness

**DOI:** 10.1007/s00122-025-04863-6

**Published:** 2025-03-11

**Authors:** Galil Tzuri, Asaf Dafna, Ben Itzhaki, Ilan Halperin, Elad Oren, Tal Isaacson, Adi Faigenboim, Yelena Yeselson, Harry S. Paris, Michael Mazourek, Joseph Burger, Arthur A. Schaffer, Amit Gur

**Affiliations:** 1 Plant Science Institute, Agricultural Research Organization, Newe Ya’ar Research Center, P.O. Box 1021, 3009500 Ramat Yishay, Israel; 2 Faculty of Agriculture, The Robert H. Smith Institute of Plant Sciences and Genetics in Agriculture, the Hebrew University of Jerusalem, Rehovot, Israel; 3 Department of Evolutionary and Environmental Biology, University of Haifa, Haifa, Israel; 4 Plant Science Institute, Agricultural Research Organization, The Volcani Center, P.O. Box 15159, 7507101 Rishon LeZiyyon, Israel; 5 Plant Breeding and Genetics, Cornell University, Ithaca, NY USA

**Keywords:** BSA-Seq, *Cucumis melo*, *Cucurbitaceae*, Fruit-quality, Genome, GWAS, Half-diallel, QTL mapping, QTLome, Sugars, Total soluble solids (TSS)

## Abstract

**Key message:**

Through meta-genetic analysis of Cucumis melo sweetness, we expand the description of the complex genetic architecture of this trait. Integration of extensive new results with published QTL data provides an outline towards construction of a melon sweetness pan-QTLome.

**Abstract:**

An ultimate objective in crop genetics is describing the complete repertoire of genes and alleles that shape the phenotypic variation of a quantitative trait within a species. Flesh sweetness is a primary determinant of fruit quality and consumer acceptance of melons*. Cucumis melo* is a diverse species that, among other traits, displays extensive variation in total soluble solids (TSS) content in fruit flesh, ranging from 2^0^ Brix in non-sweet to 18^0^ Brix in sweet accessions. We present here meta-genetic analysis of TSS and sugar variation in melon, using six different populations and fruit measurements collected from more than 30,000 open-field and greenhouse-grown plants, integrated with 15 published melon sweetness-related quantitative trait loci (QTL) studies. Starting with characterization of sugar composition variation across 180 diverse accessions that represent 3 subspecies and 12 of their cultivar-groups, we mapped TSS and sugar QTLs, and confirmed that sucrose accumulation is the key variable explaining TSS variation. All modes-of-inheritance for TSS were displayed by multi-season analysis of a broad half-diallel population derived from 20 diverse founders, with significant prevalence of the additive component. Through parallel genetic mapping in four advanced bi-parental populations, we identified common as well as unique TSS QTLs in 12 chromosomal regions. We demonstrate the cumulative less-than-additive nature of favorable TSS QTL alleles and the potential of a QTL-stacking approach. Using our broad dataset, we were additionally able to show that TSS variation displays weak genetic correlations with melon fruit size and ripening behavior, supporting effective breeding for sweetness per se. Our integrated analysis, combined with additional layers of published QTL data, broadens the perspective on the complex genetic landscape of melon sweetness and proposes a scheme towards future construction of a crop community-driven melon sweetness pan-QTLome.

**Supplementary Information:**

The online version contains supplementary material available at 10.1007/s00122-025-04863-6.

## Introduction

Melons, *Cucumis melo* L. (Cucurbitaceae), are a widely consumed, warm-season fruit vegetable. The plants are annual, heat-loving vines that thrive in warm, sunny situations. In most regions, they are planted for the production of their mature, sweet-fleshed fruits, which are highly esteemed for their quality. In *C. melo,* most plants are monoecious or andromonoecious with a flexible mating system, capable of both self-pollination and outcrossing (Rosa 1924). Fruit quality of melon is determined primarily by soluble sugar levels (Yamaguchi et al. 1977). As a whole, the Cucurbitaceae are unique in their sugar metabolism pathway: while most crop-plant families are primarily sucrose translocators, cucurbits are galactosyl-sucrose translocators (Webb and Gorham 1964; Hendrix 1982; Madore et al. 1988). Thus, the cucurbit fruit is a novel alternative model for fleshy fruit sugar accumulation and metabolism. The complex pathway of sugar metabolism in the melon fruit begins with translocated raffinose oligosaccharides and continues through multiple pathways of hydrolysis, hexose phosphorylations, transglycosylations, nucleotide sugar metabolism, sucrose cleavage and synthesis, as well as rearrangements of epimerization, isomerization and mutase reactions (Schaffer et al. 1987; Burger and Schaffer 2007; Dai et al. 2011).

The sugars occurring in mature melon fruits consist of sucrose, a disaccharide, and its two monosaccharide hydrolysis products, the hexoses glucose and fructose. Notably, the increase in total sugar content during melon fruit maturation is primarily attributable to the accumulation of sucrose during the final stages of fruit development (Rosa 1928; Elmstrom and Davis 1981; Schaffer et al. 1987, 1996; Burger and Schaffer 2007). Most importantly, sucrose levels are the main factor contributing to the genetic differences in total sugar content among *Cucumis melo* accessions (Stepansky et al. 1999; Burger et al. 2000). The accumulation of sucrose during development is determined by the genetically, environmentally and developmentally controlled metabolism that takes place within the fruit sink itself. Correlative studies of enzyme activities and sugar accumulation in melon (Gao et al. 2004; Burger and Schaffer 2007; Dai et al. 2011) have led to the observation that numerous enzymes in the pathway, including multiple isoforms of alpha-galactosidase (AGA), invertase, sucrose phosphate synthase (SPS) and sucrose synthase (SuSy), among others, show correlations between their activities and the dynamics of flesh sugar content through fruit development.

The developmental transition to sucrose accumulation was also characterized at the transcriptomic level. Comprehensive analyses of the complete pathway, comprised of over 50 genes, provided a global view of sugar metabolism gene expression during melon fruit development (Dai et al. 2011). The results of the developing transcriptome points to the same conclusion as the enzymatic studies: there is a coordinated transition of multiple components of the complex pathway responsible for the metabolic transition from sugar utilization for growth to sugar storage. Interestingly, none of these candidate genes, coding for the sugar metabolism pathway in the fruit, co-localized with any of the QTLs, known at that time, for sugar content in melon fruit (Harel-Beja et al. 2010).

There is broad genetic variability for sugar content in melon (Burger et al. 2009) and this variability can be utilized as a resource for identifying the molecular-genetic control of sugar accumulation, through a forward-genetics approach. Since 2012, the availability of reference genome sequences for melon (Garcia-Mas et al. 2012) have provided an important component for efficient translation of QTL mapping results to the discovery of candidate genes and further investigation of molecular mechanisms of traits. There is ongoing progress in studying the genetic control of the sugar-accumulation trait based on the genetic variability for sugar content in melon. Harel-Beja et al. (2010) reported six significant QTLs for sugar content based on a recombinant inbred lines (RILs) mapping population derived from the cross of the high-sugar ‘Dulce’ (subsp. *melo*, Reticulatus Group) with the low-sugar PI414723 (subsp. *agrestis*, Momordica Group). Diaz et al. (2011), who developed a consensus linkage map of melon and combined it with QTL studies from several mapping experiments, reported more than 10 QTLs for °Brix (TSS), sugars and sucrose. °Brix QTLs were reported in additional more recent mapping studies (Leida et al. 2015; Ramamurthy and Waters 2015; Zhang et al. 2016). Argyris et al. (2017) analyzed in parallel two genetic populations, between the high-sugar ‘Piel de Sapo’ (subsp. *melo*, Inodorus Group) and two different low sugar accessions, ‘Songwhan Charmi’ (subsp. *agrestis*, Chinensis Group) and “Trigonus” (subsp. *collosus*), and found that a QTL for sugar co-localized to chromosome 5 in both populations. Further fine-mapping led to the identification of a strong candidate, a BEL1-like transcription factor, for genetic control of sugar accumulation, distinguishing between the high-sugar ‘Piel de Sapo’ and the two low-sugar accessions. Pereira et al. (2018) studied a population derived from a cross between two high-sugar melons, ‘Piel de Sapo’ and ‘Védrantais’ (subsp. *melo*, Cantalupensis Group) and nevertheless were able to identify QTLs for sugar level. In two recent studies, additional QTLs were mapped for TSS. Yan et al. (2023) analyzed a RIL population segregating for sweetness, as well as a diverse collection used for a genome-wide association study (GWAS). They reported on several QTLs and proposed candidate genes within QTL intervals. Zhao et al. (2023) mapped TSS QTLs in F_2_ populations that displayed transgressive segregation compared to their Oriental melon (subsp. *agrestis*) parents. Collectively, over the years, more than 80 sweetness-related QTLs were mapped in multiple studies that represent different parental combinations across melon diversity.

Presently, we leverage a unified multi-parental framework that we recently developed for trait dissection in melon (Oren et al. 2022) to genetically analyze the variation in sugars and total soluble solids (TSS) across six different populations. Through this meta-genetic analysis, we have been able to map common as well as population-specific QTLs. We have also characterized the mode of inheritance of TSS, by analyzing a diverse, 20-parent, half-diallel (*HDA20*) population. These analyses provide a further perspective on the cumulative nature of TSS QTLs and on the genetic and physiological relationships of fruit sweetness with other traits. An ambitious standing challenge crop geneticists face is a holistic characterization of the complete repertoire of genes and alleles that shape the phenotypic variation of a quantitative trait within a species. Our objective was to gain a broader view on the genetic landscape of sweetness variation across melon diversity. For this purpose, we have integrated our own results with the results of 15 published sweetness-related QTL experiments conducted over the last 20 years in melon. This has allowed us to construct an outline for a pan-QTLome of melon sweetness.

## Materials and methods

### Plant materials and field trials

The current study was comprised of six populations that are described below and summarized together with their experimental set-ups in Table 1 and Supplementary Table S1. The parents of populations 3–6 are denominated to subspecies (subsp.) and cultivar-group (Group) and illustrated in Supplementary Fig. S1.

**Table 1:** Populations used and experiments conducted for the current study from 2015 to 2023

Set#^a^	Year	Season	Population	Population Type	Generation	Location	Traits measured	N of Genotypes	Total N Plots	Total N Plants	Total N Fruits analyzed	TSS range	TSS H^2^
1	2015	Spring	*Melo180*	GWAS	Inbred accessions	Open-Field	TSS, Sugars, AFW, DTH	177	531	2,655	2,655	3.6–16.2	0.71
2	2018	Spring	*HDA20*	Half-diallele 20	Inbred parents + F1s	Open-Field	TSS, AFW, DTH, ETE	210	630	3,150	3,150	2.4–14.1	0.78
	2019	Spring	*HDA20*		Inbred parents + F1s	Open-Field	TSS, AFW, DTH	210	630	3,150	3,150	2.3–16.2	0.82
	2020	Spring	*HDA20*		Inbred parents + F1s	Open-Field	TSS, AFW, DTH	210	630	3,150	3,150	2.9–17.2	0.80
3	2016	Spring	TAD × DUL RILs	Bi-parental	RILs	Open-Field	TSS, AFW, ETE, DTH	164	492	2,460	2,460	8.1–16.5	0.55
	2017	Spring	TAD × DUL RILs		RILs	Open-Field	TSS, AFW, ETE, DTH	164	492	2,460	2,460	10.0–16.8	0.51
	2018	Spring	TAD × DUL RILs		RILs	Greenhouse	TSS	148	740	740	740	6.5–16.1	0.57
4	2019–2021		TAD × QME F2-F5	Bi-parental	Intermediate generations F2-F5: 5 experiments and total of 4,310 plants (details in Supplementary Table S1)								
	2022	Spring	TAD × QME F6_TSS Tails		F6_Tails	Open-Field	TSS, AFW	64	128	640	640	5.0–14.6	0.78
	2023	Spring	TAD × QME F6_TSS Tails		F6_Tails	Open-Field	TSS, AFW	64	128	640	640	3.9–12.4	0.54
5	2020–2021		TAD × PI164323 F2-F5	Bi-parental	Intermediate generations F2-F5: 5 experiments and total of 2,940 plants (details in Supplementary Table S1)								
	2022	Spring	TAD × PI164323 F6_TSS Tails		F6_Tails	Open-Field	TSS, AFW	65	130	650	650	3.5–15.0	0.89
	2023	Spring	TAD × PI164323 F6_TSS Tails		F6_Tails	Open-Field	TSS, AFW	65	130	650	650	3.6–14.6	0.65
6	2016	Spring	SAS × DOYA F5	Bi-parental	F5	Open-Field	TSS	117	234	1,170	1,170	3.0–13.2	0.80
	2022	Spring	SAS × DOYA F5_TSS Tails		F5_Tails	Open-Field	TSS, AFW	60	120	600	600	3.7–14.9	0.84
	2023	Spring	SAS × DOYA F5_TSS Tails		F5_Tails	Open-Field	TSS, AFW	60	120	600	600	2.3–16.5	0.86

*TSS* total soluble solids, *DTH* days to harvest, *AFW* average fruit weight, *ETE* ethylene emission

^a^Germplasm set # as in the Materials and methods

(1) *Melo180* panel—The Newe Ya’ar melon collection used in this study was comprised of 177 diverse accessions (Gur et al. 2017). Almost all of these accessions derive from the two cultivated subspecies, *melo* and *agrestis* (Deleu et al. 2009; Serres-Giardi and Dogimont, 2012), which together encompass 16 cultivar-groups (Pitrat et al. 2000; Burger et al. 2009); a few are derived from the feral melon, subspecies *collosus*. Each accession was represented by three plots of five plants in a randomized complete block design (RCBD) in an open field experiment at the Newe Ya’ar Research Center, northern Israel (32° 43′ 05.4″ N 35° 10′ 47.7″ E) in spring–summer 2015.

**(2)** *Core25* set, *HDA25F*_*2*_ library and the *HDA20* half-diallel population—A core set of 25 diverse melon accessions was selected based on a comprehensive genotypic and phenotypic characterization of our broader *Melo180* panel (Gur et al. 2017). The accessions for this core set were selected based on the multiple genotypic and phenotypic criteria previously described (Gur et al. 2017; Dafna et al. 2021). The creation of the diverse, 25-way half-diallel population (*HDA25*), resulting in 300 F_1_ hybrids that represent all possible parental combinations, was previously described by Dafna *et al*. (2021). *HDA20*, a subset of the *HDA25* set, is composed of 20 representative accessions of the 25 members of the *Core25*. The 20-way half-diallel population of *HDA20* resulted in 190 F_1_s. The 190 *HDA20* F_1_s and their 20 parents were grown in the open field at Newe Ya’ar in three field experiments during the summer seasons of 2018, 2019 and 2020. Each genotype was planted in three plots of five plants each in an RCBD setup. All 300 *HDA25* F_1_s were grown in a greenhouse at Newe Ya’ar and subjected to self-pollinations to produce 300 F_2_ populations, previously defined as the *HDA25F*_*2*_ library (Oren et al. 2022), and were used in the current study to select and advance the segregating populations.

**(3)** ‘Tam Dew’ (TAD) × ‘Dulce’ (DUL) RIL population (*HDA034*F_7_) – A bi-parental population of 164 F_7_ recombinant inbred lines (RILs) was developed through single seed descent from a cross between ‘Tam Dew’ (TAD; subsp*. melo*, Inodorus Group) with sweet large round fruits and smooth rind, and ‘Dulce’ (DUL; subsp*. melo*, Reticulatus Group), a sweet, medium-size, round, heavily netted American muskmelon (Tzuri et al. 2015). All RILs, F_1_ and parental accessions were grown in an RCBD in the summer seasons of 2016 and 2017, in the open field at the Newe Ya’ar Research Center. Each line was represented by two replicates of five plants per plot. In the spring–summer season of 2018, the population was grown in a 50-mesh net-house in an RCBD, at Newe Ya’ar; soil type was a standard planting mixture, and the plants were drip-irrigated and drip-fertilized. Each line was represented by five replicates of a single plant. In all trials, the parental accessions and F_1_ were grown in five replicates randomly distributed.

**(4)** ‘Tam Dew’ (TAD) × Qishu Meshullash (QME) F_6_ population (*HDA008*F_6_) – ‘Tam Dew’ (TAD; subsp. *melo* Inodorus Group) was crossed with a non-sweet accession named “Qishu Meshullash” (QME, subsp. *collosus*), in a greenhouse at Newe Ya’ar and 218 F_2_ progenies were advanced to the F_6_ generation by the single-seed-descent selfing scheme. Qishu Meshullash was reported to have been collected from a feral population growing in Israel and was obtained from the Israel Gene Bank. We identified it as belonging to subsp. *collosus* based on the recent clarifications of this taxon (John et al. 2013; Endl et al. 2018). During population development, 1–2 mature fruits per plant were sampled from each of the lines in each generation (F_2_–F_5_) for TSS measurements used for selective phenotyping. Extended tails of 33 high and 34 low TSS F_6_ lines were selected for validation and bulks construction for QTL-Seq.

**(5)** ‘Tam Dew’ (TAD) × PI164323 (*HDA192*F_6_) population—150 F_2_ progenies of the cross between the sweet-fleshed ‘Tam Dew’ and the non-sweet accession PI164323 (subsp. *melo*, Adzhur Group) were advanced to the F_6_ generation by the single-seed-descent selfing scheme. During population development in the greenhouse at Newe Ya'ar, 1–2 mature fruits per plant were sampled from each of the lines in each generation (F_2_–F_5_) for TSS measurements used for selective phenotyping. Extended tails of 25 high and 25 low TSS F_6_ lines were selected for validation and bulk construction for QTL-Seq.

**(6)** ‘Sakata's Sweet’ (SAS) × Doya's Faqqous (DOYA) (*HDA243*F_5_) – 117 F_2_ progenies from the cross between the sweet-fleshed ‘Sakata's Sweet’ (subsp. *agrestis*, Makuwa Group) and the locally grown, non-sweet accession that we named “Doya's Faqqous” (subsp. *melo*, Flexuosus Group) were advanced to the F_5_ generation by the single-seed-descent selfing scheme. Selective phenotyping was based on the TSS data collected on the F_5_ population in a replicated field trial conducted during the summer season of 2016 at Newe Ya’ar. Extended tails of 21 high and 27 low TSS F_5_ lines were selected for validation and bulk construction for QTL-Seq.

Selected subsets of the three advanced generations defined as QTL-Seq populations (*HDA008*F_6_-tails, *HDA192*F_6_-tails, *HDA243*F_5_-tails) were phenotyped for TSS over two years (2022 and 2023) alongside their parental accessions and corresponding F_1_ hybrids. The extended tails from the early-generation TSS distributions were phenotyped in replicated open-field experiments at Newe Ya’ar, each of the selected lines was represented by 10 plants that were arranged in two randomized blocks.

### TSS and sugar analysis

Five mature fruits per plot were sampled in multiple harvests three times a week (over 3–4 weeks) to match the accurate maturity timing of each entry. Fruits were harvested at maturity based on abscission, fruit softening or rind color in climacteric fruits, or rind color and days after fruit set (45–50 days) in non-climacteric fruits. A total of 10–15 fruits per accession were analyzed in each experiment. Concentrations of total soluble solids (TSS, expressed in °Brix) were measured using a hand-held refractometer (Atago, Tokyo, Japan). Cylindrical flesh samples were taken from the center of the fruit equator from each of the five fruits separately (Supplementary Fig. S2). The internal seed coating gel and connecting tissue (placenta) were removed from each cylinder and the samples were squeezed against a sieve to extract juice for the refractometer measurements. Approximately 1 g of mesocarp tissue, taken from the center-equatorial portion of the fruit, was placed in 15 ml tubes with 80% EtOH, and soluble sugars (glucose, fructose and sucrose) were extracted and analyzed by high-performance liquid chromatography as previously described (Petreikov et al. 2006).

### Statistical analyses

The JMP ver. 14.0.0 statistical package (SAS Institute, Cary, NC, USA) was used for all the general statistical analyses (including frequency distributions, correlations, analyses of variance and mean comparisons). Broad-sense heritability (*H*^*2*^ = (Var (G)/Var (P)) was estimated in each experiment separately using analysis of variance (ANOVA) based variance components. In mode of inheritance analyses: the additive effect (*a*) was calculated as half of the difference between parental lines. The dominance effect (*d*) is the difference between the F_1_ hybrid and the mid-value of its parents. The degree of dominance (*d*/[*a*]) was calculated by dividing dominance value by the additive effect. In genome-wide QTL analyses, the significance threshold was corrected for multiple comparisons using the FDR approach (Benjamini and Hochberg 1995).

### DNA preparation, SNP genotyping and whole-genome sequencing

DNA isolations were performed using the GenElute™ Plant Genomic Miniprep Kit (Sigma-Aldrich, St. Louis, MO, USA). DNA quality and quantification were determined using a Nanodrop ND-1000 (Nanodrop Technologies, Wilmington, DE, USA) spectrophotometer, electrophoresis on an agarose gel (1.0%), and Qubit® dsDNA BR Assay Kit (Life Technologies, Eugene, OR, USA).

Genotyping of the TAD × DUL RIL population was performed using genotyping by sequencing (GBS), and map construction was as previously described by Oren et al. (2020). Map construction was based on 89,343 SNPs across 146 lines. Originally, SNPs were mapped to the DHL92V3.5.1 genome and were later re-mapped to the latest version DHL92V4.0 using the Genome Analysis ToolKit (GATK) (Broad Institute, Cambridge MA, U.S.A.). SNP filtration was done with TASSEL v.5.2.43 (Bradbury et al. 2007) and linkage-map construction was done using the ASMap R package (Taylor and Butler 2017) [https://doi.org/10.18637/jss.v079.i06.]. Genotyping of the *Melo180* diversity panel was performed using GBS, as described by Gur et al. (2017) and the final SNP set included 23,931 informative SNPs across 177 accessions. Whole-genome sequencing, de novo assembly and SNP and InDel discovery across the *Core25* set were as described in detail by Oren et al. (2022).

Whole-genome sequencing of gDNA of differential TSS bulks was performed by Syntezza Bioscience (Jerusalem, Israel). For each sample, 200 ng of total genomic DNA were fragmented with Covaris-S220 for an average fragment size of 300 bp. For library preparation, the xGen Prism DNA Library Prep Kit (IDT) was used, and 250 ng of each library were sequenced by Novaseq 3000 (PE150, 12 GB/sample).

### *QTL mapping in the Tam Dew* × *Dulce RIL population*

QTLs were analyzed as previously described (Oren et al. 2020). In brief, TASSEL ver. 5.2.51 (Bradbury et al. 2007) was used for genome-wide linkage analysis of the traits using a generalized linear model (GLM) with 1,000 permutations and a *P* value of 0.05 as the threshold for significance.

### GWAS analysis on the Melo180 panel

Association analysis for TSS on the *Melo180* diverse collection was performed with TASSEL v.5.2.51 (Bradbury et al. 2007) as described in detail by Gur et al. (2017). Briefly, a mixed linear model (MLM) using both population structure (Q-matrix) and relatedness (kinship matrix) was used for statistical analysis of genome-wide associations. This model considered population structure and cryptic relationships, thereby minimizing false positives and increasing the statistical power. Additionally, we used a naïve generalized linear model (GLM) without any consideration for population structure, as a means to observe and compare the non-corrected pattern of *P* values.

### Construction of three TSS mapping populations and selective phenotyping of tail-segregants

Considering the limitations of GWAS for mapping QTLs for a complex trait such as TSS, we decided to construct and use multiple bi-parental segregating populations for QTL mapping analysis. Phylogenetic analyses of *Cucumis melo* supported the division of the species into two major cultivated subspecies, *agrestis* and *melo* (Kirkbride 1993; Deleu et al. 2009; Serres-Giard and Dogimont 2012), which together comprise about 16 cultivar-groups (Pitrat et al. 2000). The difference in number and identity of genes determining sugar levels that are polymorphic in different crosses is very likely a reflection of the genetic distance and evolutionary history of the different cultivar-groups. Based on this we have identified pairs of low and high sugar accessions, which represent different parental combinations. Following the scheme described by Oren et al. (2022), we first identified three relevant crosses between TSS-differential parental accessions from our *Core25* set. We used a common sweet parent, ‘Tam Dew’ (TAD, °Bx = 15.2), which is a non-climacteric honeydew melon from the Inodorous Group (subsp. *melo*), for two crosses. The non-sweet parents for these populations are from two different subspecies: Qishu Meshullash (QME, °Bx = 8.0)—a small-fruited feral accession of subsp. *collosus,* and PI164323 (°Bx = 4.9)—an elongate-fruited melon classified within the Adzhur Group of subsp. *melo* (Supplementary Fig. S3a). The third cross was inter-sub-specific, the sweet parent being ‘Sakata's Sweet’ (SAS, °Bx = 15.0), from subsp. *agrestis*, Makuwa Group, and the non-sweet parent being Doya's Faqqous (DOYA, °Bx = 3.8), from subsp. *melo*, Flexuosus Group (Supplementary Fig. S3a). From each of these three crosses, we obtained F_2_ populations from our *HDA25F2* library that represents all 300 combinations of the 25-parent half-diallel. We advanced these three selected populations through the single-seed-descent (SSD) scheme and TSS data were collected in each generation as decision-supportive data. To implement the 'selective phenotyping' strategy for QTL-Seq mapping, we used the early-generation TSS correlations and selected extended-tail segregants that represent extreme contrasts for TSS in the TAD × QME and TAD × PI164323 populations (Supplementary Fig. S3b, c). For the SAS × DOYA population, we used TSS correlation between replications in the F_5_ generation to select tail segregants (Supplementary Fig. S3d). The selected extended tails from the three populations (*n* = 180) were then phenotyped for TSS as F_5_ and F_6_ lines in a replicated field experiment in the summer of 2022. Based on this validation, several lines were excluded, and 143 confirmed extreme lines were used for the QTL-seq analysis (Supplementary Fig. S3f, g, h and. Supplementary Fig. S4).

### Bulk segregant analysis by sequencing (QTL-Seq)

DNA samples from the selected high- and low-TSS tail segregants from the three F_5_-F_6_ populations were prepared in two bulks for each population. These samples were used for whole-genome resequencing (WGS) performed by Syntezza Bioscience to an average depth of ~ 30 × . All raw reads were mapped to the melon reference genomes, DHL92 v4.0 (Castanera et al. 2020) using the Burrows–Wheeler Aligner (BWA), producing analysis-ready BAM files for variant discovery with the Broad Institute’s Genome Analysis Toolkit (GATK) (McKenna et al. 2010). Polymorphic sites were extracted from the vcf file that was further filtered to a total depth of > 20 reads per site. The read-depth information for the SNPs in the high- and low-TSS bulks was obtained to calculate the SNP-index (Takagi et al. 2013). For each site, we then calculated within each bulk the ratio of the number of ‘reference’ reads to the total number of reads, which represented the SNP-index of that site. The difference between the SNP-index of high- and low-TSS pools in each population was calculated as ΔSNP-index. The sliding window method was used to draw the ΔSNP-index trend line. Candidate genomic regions associated with TSS were defined surrounding a ΔSNP-index ≥ 0.4. We performed whole-genome scans to identify genomic regions with significant ΔSNP-index signals.

### 'Bulk-opening' and validation of QTL-Seq signals using PCR markers

To validate the QTLs detected through bulk sequencing analysis and to obtain detailed descriptions of the effects and percent variation explained by each QTL, we developed specific PCR markers for QTL peaks (Supplementary Table S2) and genotyped all the lines that composed the bulks in the three populations. To identify relevant polymorphic sites at the QTL peaks within each of the parental pairs, we used the genome-wide InDel database that we previously constructed through the 300 pairwise genome comparisons of all *HDA25* parental combinations using the de novo genome assemblies of the *Core25* panel (Oren et al. 2022). This database includes 115,802 short InDels, filtered to a size range of 10–2,000 bp. Based on the physical positions of these InDels on the database, for each QTL we searched for polymorphic InDels at the peak and at the two ends of the confidence interval. Raw genome sequences were then reviewed in these regions using the Integrative Genome Viewer (IGV) (Robinson et al. 2011) and only InDels that displayed clean presence/absence of sequence reads that matched the predicted InDel profile were analyzed further (Supplementary Fig. S5). Primers were designed flanking InDel intervals and were first tested on the parental accessions and F_1_s of the populations. PCR products were run on 2.5% agarose gel. Validated InDels, showing clear co-dominant polymorphisms, were used to genotype all the high- and low-TSS selected lines from the three populations. These genotypic data allowed us to calculate more informative quantitative descriptors of QTLs, such as their allelic effects, percent variation explained (PVE) and significance based on analysis of variance (ANOVA) performed within each population. It is important to note that allelic effects and PVE values may be inflated due to the use of extreme tail segregants, but on the other hand, *P* values are underestimated due to the smaller number of degrees of freedom (*df*) used in the statistical analysis.

### Integrated sweetness-related QTL map construction

In addition to the 23 QTLs mapped in the current study, we extracted QTL information from 15 additional experiments from 2004 until 2023. In all the studies conducted until 2012, before the publication of the first melon reference genome (Garcia-Mas et al. 2012), QTLs were positioned onto population-specific linkage maps. Diaz et al. (2011) constructed a consensus linkage map where QTLs from several studies were aligned to a common map using anchor markers. In some of the studies conducted after the publication of the melon reference genome, QTL positions are reported in physical coordinates of earlier genome versions. To align all the past QTLs to the latest assembly of the DHL92 reference genome (Version 4.0, Castanera et al. 2020) we BLASTed sequences of QTL flanking markers or sequences at physical positions reported in earlier versions, against the DHL92V4.0 assembly, and the output positions are used here as the aligned QTL positions (Supplementary Table S5).

Comparative analysis of QTLs across genomic bins was performed by partitioning the genome to 5 Mb bins and counting the number of QTLs that fall within each of the defined bins across the 20 studies. Large QTL intervals (> 15 Mb) were excluded from this analysis.

## Results

### Characterization of TSS and sugar composition in the Melo180 panel

**Fig. 1:**
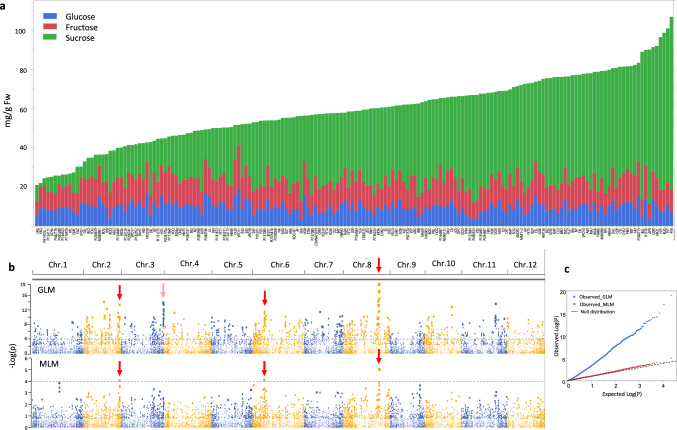
Characterization of TSS and sugar composition and GWAS of TSS in the Melo180 diverse collection. **a** Sugar profile in flesh of mature fruits of 177 diverse accessions. Accessions are ordered by their total sugars. **b** Manhattan plot of GWA results for TSS. Upper pane is based on GLM analysis and displays the genome-wide distribution of naïve model P values. Lower pane is MLM with population relatedness correction. Dashed horizontal line is the significance threshold. Red arrows indicate significant QTL peaks identified in the MLM analysis with a visible association signal (above the false-discovery noise) also in the GLM analysis. Pink arrow on chr.3 is a significant association identified only by the GLM analysis. **c** Quantile–quantile (Q-Q) plot for distribution of TSS P values in the MLM model. The negative logarithm of the observed (y-axis) and the expected (x-axis) P values are plotted for each SNP (dot). The gray dashed line indicates the null hypothesis

Beginning with characterization of TSS and sugar composition variation across the *Melo180* population, with its 177 diverse accessions containing representatives of 3 subspecies of *Cucumis melo* (the cultivated subspp. *melo* and *agrestis*, and the feral subsp. *collosus*) and 12 of the cultivar-groups (8 from subsp. *melo*, 4 from subsp. *agrestis*), we observed a moderate to high broad-sense heritability of TSS across this diverse germplasm (*H*^2^ = 0.71). Quantification of sucrose, glucose and fructose in the mature fruit flesh reconfirmed that variation in total sugar content (and its correlative descriptor, TSS) is mostly explained by variation in sucrose content, while there is minor variation in the content of the monosaccharides, glucose and fructose (Fig. 1a, Supplementary Fig. S6). Sucrose ranged from 1–107 mg/gfw and TSS ranged from °Brix 4–16, expressing the wide variation for fruit sweetness across *C. melo* diversity (Supplementary Fig. S7). Projection of TSS on the genetic PCA plot, constructed based on 23,900 SNP markers, shows that overall high-, intermediate- and low-TSS accessions are distributed across the diversity of both cultivated subspecies, including presence of sweet and non-sweet accessions in both (Supplementary Fig. S7). This could indicate that multiple, different genetic factors are contributing to sweetness in different genotypes. We then used the 23,900 SNPs to conduct genome-wide association analysis (GWA, Fig. 1b, c). The extensive phenotypic variation and significant heritability of TSS across *Melo180* were not expressed in the number and magnitude of QTLs in a relatedness-corrected mixed linear model (MLM). Only three significant moderate effect QTLs were mapped to chromosomes 2, 6 and 8 (Fig. 1b, lower panel). Interestingly, in the naïve (GLM) model (upper panel), we identified a strong association signal (above the false-discovery noise) also on chromosome 3, which was not significant in the relatedness-corrected analysis (Fig. 1b). This QTL also had a significant effect on sucrose contents (Supplementary Fig. S8) and it is highlighted here since it is described further, below, as a common QTL across multiple bi-parental populations. The lack of significance of this QTL in the MLM analysis is likely due to correlation of causative allelic variation in this locus with population structure and relatedness matrix.

### Mode of inheritance of TSS in the HDA20 half-diallel population

**Fig. 2:**
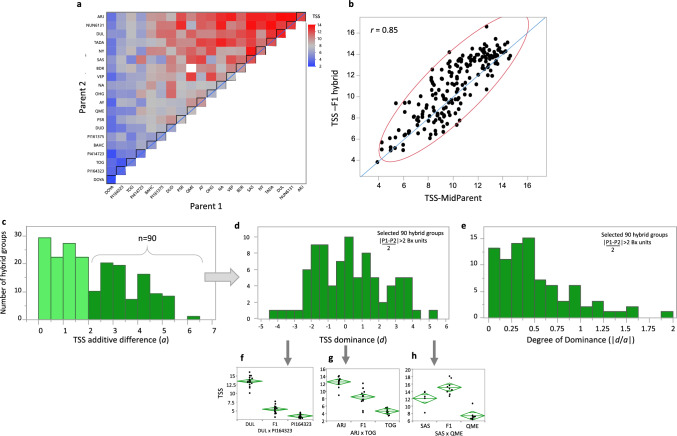
Mode-of-inheritance of TSS in 190 half-diallel F_1_ hybrids and their 20 parents. **a** Heat map of the 20 × 20 half-diallel matrix for TSS. Both axes are ordered by parental GCA, which was based on the mean of all 19 crosses for each parent. Diagonal is the performance per se of each of the parents. **b** Correlation between parental mean (mid-parent) and F1 hybrid for TSS across 190 hybrid groups (HDA20). Red ellipse indicates the bivariate normal density at 95% coverage. **c** Frequency distribution of additive TSS difference, *a*, across 190 half-diallel parental pairs; the 90 hybrid groups with *a* > 2 are highlighted; these were selected for the dominance analysis. **d** Frequency distribution of TSS dominance effect, *d*, across 90 selected hybrid groups with *a* > 2; *d* is the difference between the F1 and its parental mean. **e** Frequency distribution of degree of dominance (*d/a*) across 90 selected hybrid groups with *a* > 2. **f** Example of dominant mode-of-inheritance towards low-TSS in the DUL × PI164323 hybrid group. **g** Example of complete additivity (no dominance deviation) in the ARJ × TOG hybrid group (members of the Core25 set). **h** Example of over-dominant mode-of-inheritance towards high-TSS in the SAS × QME hybrid group. In **f–h**, mean diamonds show the trait mean and confidence interval. Mid-horizontal line is the trait mean. The top and bottom of each diamond span the 95% confidence interval for the mean of each group. The lines near the top and bottom of the diamonds are overlap marks for visual statistical difference between group means

In order to characterize the mode-of-inheritance of TSS across melon diversity, we analyzed the half-diallel population that we previously constructed (Dafna et al. 2021) by intercrossing 20 diverse core lines in all possible combinations, resulting in 190 F_1_ hybrids (*HDA20*). This set of hybrids and their parents was phenotyped in replicated field trials over three years. High correlations between years on an accession-mean basis was observed (*r* = 0.84–0.88, *n* = 210), supporting the high heritability of TSS (Supplementary Fig. S9). A simple, visual way to observe the TSS variation and inheritance pattern is by plotting genotype means on a 20-parent half-diallel heatmap, where each cell represents an F_1_ by its parental combination and diagonal cells represent the parents themselves (Fig. 2a). By sorting this matrix on both axes from low to high TSS, the additive mode of inheritance is reflected by the non-random transition from blue to red along the diagonal and rows and columns. Another simple view of the robust additive inheritance pattern of TSS is by looking at this population as a set of 190 hybrid groups, where each group is composed of an F_1_ and its two parents. For each hybrid group, the parental mean (mid-parent) TSS is plotted against the F_1_ TSS; the high correlation coefficient (*r* = 0.85, Fig. 2b) is the result of a strong additive component. Deviations from the x = y diagonal in Fig. 2b represent the dominance deviations of F_1_s from the pure additive value. To characterize further the dominance component in TSS inheritance, we selected 90 hybrid groups where the additive (*a*) difference between parental lines was > 2°Brix units (Fig. 2c). Analysis of the dominance effects across this differential subset showed a normal distribution with deviations from additivity towards both high- and low-TSS parents, and a mean not different from zero (*d* = 0.28, Fig. 2d). The average (absolute) degree of dominance was *d/a* = 0.51 across this set of 90 differential hybrid groups (Fig. 2e), indicating a contribution of a dominance component to hybrid TSS performance in crosses of parents differing in their fruit sweetness. An example of hybrid groups where F_1_s displayed significant dominance deviation towards low-TSS parents is provided in Fig. 2f, little or no deviation with *d* = 0 in Fig. 2g, and with significant dominance deviation towards high-TSS parents, even over-dominance, in Fig. 2h.

### Mapping TSS QTLs in four bi-parental populations

**Fig. 3:**
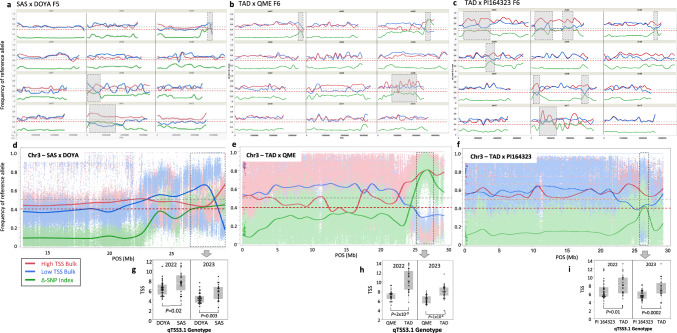
Bulk-sequencing analysis (BSA-Seq) of three sweet × non-sweet melon populations. **a-c** Whole-genome view of BSA-Seq results of the high- versus low-TSS bulks in the SAS × DOYA F5 population (**a**), TAD × QME F6 (**b**) and TAD × PI164323 F6 (**c**). The 12 melon chromosomes are presented. Physical positions are based on the DHL92 v4.0 genome. Trend-lines are the running average of SNP-Index and Δ-SNP Index values. Blue line is the SNP-index in the low-TSS bulk. Red line is the SNP-index in the high-TSS bulk. Green line is the Δ-SNP Index. Dashed red horizontal line is the 0.4 threshold for significance of the Δ-SNP Index. **d-f** Zoom in on chromosome 3 for BSA-Seq results of the high- versus low-TSS bulks in the SAS × DOYA F5 population (**d**), TAD × QME F6 (**e**) and TAD × PI164323 F6 (**f**). Background dots are the SNP Index and Δ-SNP Index at each of the more than ~ 250,000 informative SNPs along the chromosome. Red = High-TSS bulk, Blue = Low-TSS bulk, Green = Δ-SNP Index. Dashed red horizontal line is the 0.4 threshold for significance of the Δ-SNP Index. Dashed rectangle is the QTL interval in each populations. **g-i** Allelic effect analysis at the qTSS3.1 QTL-peak marker in 2022 and 2023. Homozygote genotypes are presented. Each point represents the mean of an F5 or F6 line

We performed QTL mapping by whole-genome resequencing of DNA bulks in the three bi-parental populations to an average read depth of ~ 30 × in each bulk. In the SAS × DOYA F_5_ bulks, we identified 6.8 million polymorphic sites and after quality and depth filtrations, a set of 3.3 million high-quality SNPs was defined. Comparison of allele frequencies between the high- and low-TSS bulks across the 3.3 million genome-wide loci (ΔSNP-index analysis) revealed three genomic regions (chromosomes 3, 8, 11) with significant effects in this population (Fig. 3a). In the TAD × QME F_6_ bulks we filtered 3.5 million informative SNPs and through ΔSNP-index analysis identified significant effects on chromosomes 1, 3 and 9 (Fig. 3b). In the TAD × PI164323 F_6_ bulks, we identified 3.1 million informative SNPs. ΔSNP-index analysis resulted in mapping of significant effects in seven genomic regions (Chromosomes 1, 2, 3, 4, 8, 11) in this population (Fig. 3c). To validate these QTLs, we 'opened the bulks' and genotyped all the individual extreme segregant lines that composed them, using InDel-based PCR markers that we developed in the QTL-peaks regions from the parental genome sequences (Supplementary Table S2). In addition to the validation, this analysis allowed us to calculate more informative quantitative descriptors of QTLs, such as their allelic effects, percent variation explained (PVE) and significance based on analysis of variance (ANOVA) (detailed in Supplementary Table S3 and methods). Further validation for the mapped QTLs was performed by phenotyping all 180 selected extreme lines for TSS in a second year field experiment (Supplementary Table S3). Notably, in the field experiment of 2023, all sweet accessions across the different experiments did not accumulate as much sugar as in most years (Supplementary Fig. S3f-h) and, consequently, while correlation between years was high (Supplementary Fig. S10), QTL effects in this year were generally lower (Supplementary Table S3). Collectively, we identified through the QTL-seq analyses, 7 population-specific QTLs and 5 common QTLs. A consistent QTL that was mapped in these three populations, and confirmed over two years, is *qTSS3.1* on chromosome 3 (Fig. 3 d-i).

**Fig. 4:**
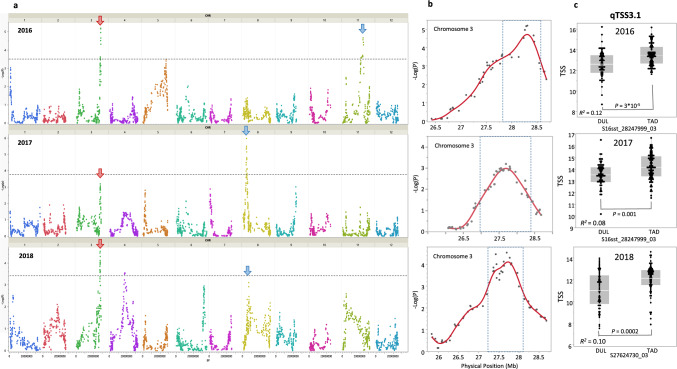
TSS QTL mapping in the TAD × DUL RIL population over 3 years. **a** whole-genome QTL analysis over three years (2016–2018). Dashed horizontal lines are the genome-wide QTL significance threshold. Red arrows indicate qTSS3.1 over 3 years. Blue arrows are non-consistent QTLs. **b** Chromosome 3 QTL plot in 2016 and 2018. The dashed rectangle indicates the QTL borders based on a 1.5 LOD confidence interval. **c** Allelic effect analysis at the qTSS3.1 QTL-peak marker in 2016–2018. Homozygote genotypes are presented. Each point represents the mean of a RIL in the TAD × DUL population

In addition to the above three populations constructed for bulk-sequencing analyses, we used the TAD × DUL RIL population that was previously genotyped with 89,343 GBS SNPs (Oren et al. 2020) as a fourth population for TSS QTL mapping. Both TAD and DUL are sweet melon lines, but they originate from different cultivar-groups within subsp. *melo* (Inodorus and Reticulatus, respectively). The RIL population was phenotyped for mature fruit TSS over three years, and interestingly, we observed significant transgressive segregation extending the population TSS variation beyond the parental range in all the experiments (Supplementary Fig. S3e). We identified three QTLs (chromosomes 3, 8, 11) that passed the false discovery rate (FDR) significance threshold in at least one year (Fig. 4a). The chr.3 QTL was the most significant locus with consistent effect in all experiments (Fig. 4b, c). The favorable, TSS-increasing allele in this QTL was contributed by TAD and explained 8–12% of the TSS variation (Fig. 4c).

### Integrative broad perspective on TSS and sugar QTLs in melon

**Fig. 5:**
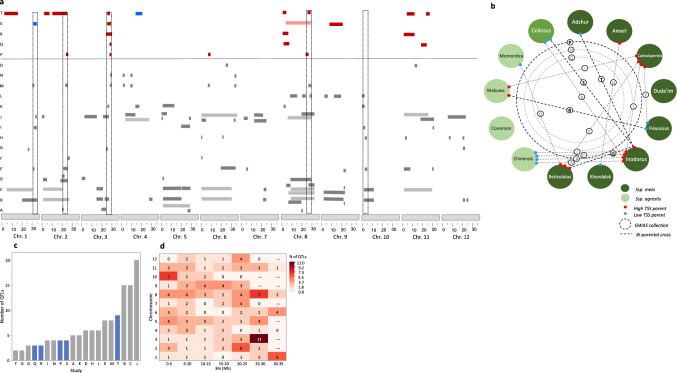
Sweetness-QTLome: Integrative TSS QTL mapping across 5 populations in the current study alongside sugars and TSS QTLs mapped in 15 previous experiments in melon. **a** Comparative map plotting sweetness-related QTLs across 20 experiments. Each row represents an experiment (A–T, details on each population and experiment are provided in Sup. Table S4). The 12 melon chromosomes with their physical map coordinates (Mbp) are plotted on the bottom. Rectangles represent the positions of QTLs. Very thin rectangles mostly represent significant GWAS markers without defined QTL confidence intervals. Rows P–T in the upper part are QTLs mapped in the current study. Red rectangles are significant QTLs where the favorable (high TSS) allele is contributed by the high-TSS parent. Blue rectangles are significant QTLs where the favorable (high TSS) allele is contributed by the low-TSS parent. Gray horizontal rectangles in rows A–O are a collection of sweetness QTLs reported in 15 published experiments. Light colored rectangles (gray and pink) are large QTLs that span more than 15 Mbps and excluded from the bins analysis. Dashed vertical rectangles represent chromosomal bins with QTLs in 6 or more studies. **b** Overview of the diverse melon germplasm used for the integrated TSS QTLome. Each circle represents a cultivar-group within Cucumis melo. Letters attached to dashed lines or circle represent the corresponding population/experiment as listed in Sup. Table S4. **c** Bar chart for the number of QTLs per population (across the 20 studies described in Sup. Table S4). Gray bars represent previous studies. Blue bars are populations in the current study. **d** Heatmap for the number of QTLs within 5 Mb chromosome bins across the 20 studies

To obtain an integrative view of the TSS QTLs that we mapped across five populations, we generated a comparative map, using the melon reference genome (DHL92V4.0) as the common anchor (Fig. 5a, Lanes P–T). Collectively, across the 5 populations analyzed we identified 12 genomic regions associated with TSS in one or more populations. Five QTLs were significant in two or more populations and the most significant and consistent QTL (*qTSS3.1*) was mapped to chromosome 3 with a common peak at 26.5–28Mb (Figs. 3, 4, 5). In the TAD × DUL RIL population, this QTL was defined to an interval of ~ 500 Kb (Fig. 4b). In the SAS × DOYA F_5_ tails, *qTSS3.1* explained 23% and 25% of the variation in 2022 and 2023, respectively (Supplementary Table S3). In the TAD × QME F_6_ tails, this QTL explained 47% and 35% of the variation in the two years (Supplementary Table S3). Consistent with the multiple TSS QTLs identified in the TAD × PI164323 F_6_ tails, *qTSS3.1* explained 10% and 22% of TSS variation in 2022 and 2023, respectively. Interestingly, *qTSS3.1* also consistently explained 8–12% of the TSS variation also in the sweet × sweet, TAD × DUL population (Fig. 4).

To expand further our perspective on sweetness-related QTLs across melon diversity, we added to the integrated-map QTL information from 15 additional experiments published from 2004 to 2023 (Fig. 5, Supplementary Tables S4 and S5). With all the necessary cautions and limitations of low QTL mapping resolutions, such analysis provides an important, broader view on the overall genetic landscape of QTLs affecting melon sweetness. Including the current study, at least 132 sweetness-related QTLs were reported across 20 different experiments that represent different population structures and varying combinations of parental accessions across *Cucumis melo* groups (Fig. 5b, c). Specifically, 6 out of the 12 genomic regions of QTLs in the current study represent overlap of QTL intervals in at least 4 experiments (Fig. 5a). Four of them (Chromosomes 1, 2, 3 and 8) represent overlap of 6 or more QTLs. Only a single QTL (qsweet.T.1.1, at ~ 10Mb on Chr. 1) was mapped to a genomic region that did not overlap with any other reported narrow QTLs. On the other hand, in the current study we did not identify QTLs within a common QTL interval that was reported in seven previous studies on chromosome 10 (Fig. 5a, d). This suggests that the causative allelic variation in this QTL was not present in our populations. The 20 QTL mapping populations summarized for this comparative analysis represent good coverage of bi-parental combinations and diversity panels. Together, they capture a large proportion of the *C. melo* diversity that is relevant for segregation of sweetness-related traits (Fig. 5b, Supplementary Tables S4). Figure 5c plots the number of QTLs within each population that ranges from 2 to 20 with an average of 6.6 QTLs per study. The presence of QTLs within the defined 5 Mb chromosome bins across studies ranges between 0 and 11 (Fig. 5d). In 15% of the bins (11 out of 75) no QTLs are located, and in 37% of the bins (28 out of 75) QTLs were identified in 3 or more studies.

### Interactions and cumulative effects of TSS QTLs

**Fig. 6:**
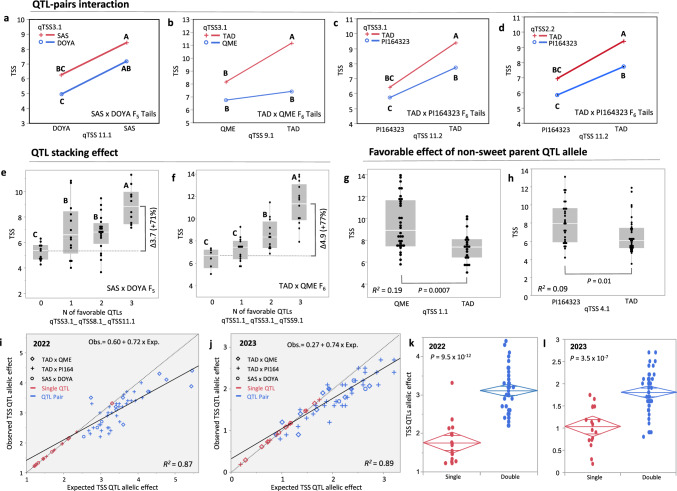
Cumulative nature of TSS QTLs. **a–d** Analyses of QTL-pair interactions across three populations. Values not connected by a common letter are significantly different at P < 0.05. **e–f** triple-QTL stacking effect in the SAS × DOYA F5 population (**e**) and TAD × QME F6 population (**f**). **g–h** Allelic effect analysis for qTSS1.1 and qTSS4.1 where the favorable, TSS-increasing allele is contributed by the low-TSS parent. **i–j** Correlation between expected and observed TSS of QTL pairs across three populations in 2022 and 2023. Dashed diagonal is the expected = observed line. Continuous regression line is shown for both years and its linear function is presented. **k–l** Comparison of allelic effects of single QTLs versus QTL-pairs in 2022 and 2023. Mean diamonds show the trait mean and confidence interval. Mid-horizontal line is the trait mean. The top and bottom of each diamond span the 95% confidence interval for the mean of each group. The lines near the top and bottom of the diamonds are overlap marks for visual statistical difference between group means

We used the 180 tail segregants to evaluate the patterns of interactions and additivity between QTLs within populations. Pairwise interactions between the major QTLs in each cross were analyzed (Fig. 6a-d). For example, analysis of *qTSS3.1* and *qTSS11.1* in the SAS × DOYA population showed complete additivity, such that the combined QTL-pair genotype represents the sum of independent effects (Fig. 6a). A similar pattern was observed across the other pairs, with an indication for a more-than-additive epistatic interaction in the qTSS3.1/qTSS9.1 QTL combination in the TAD × QME population (Fig. 6b). We further provide examples for triple QTL-stacks where pyramiding of three QTLs significantly improved the cumulative effect of two QTLs, irrespective of the combination. In the SAS × DOYA population, stacking of favorable alleles at *qTSS3.1*, *qTSS8.1* and *qTSS11.1* improved TSS by 3.7 Bx units (71%) compared to the non-favorable haplotype in these QTLs (Fig. 6e). In the TAD × QME population, stacking of favorable alleles at *qTSS1.1*, *qTSS3.1* and *qTSS9.1* significantly improved TSS by 4.9 Bx units (77%), as compared to the non-favorable reference haplotype (Fig. 6f). Statistical confirmation for the robustness of the QTL stacking effect across the three populations is provided in Fig. 6k, l, where genotypes with favorable alleles at two QTLs (double) had significantly higher TSS compared to single QTLs.

To provide a broader view on the mostly additive (non-epistatic) pattern between QTLs across the three populations, we first calculated the expected, non-epistatic, effects of 47 QTL-pairs as the sum of their singular effects. The expected effects were then plotted against the actual observed performance of these QTL-pairs, over two years (Fig. 6i, j). A significant linear relation was found between observed and expected (*R*^*2*^ = 0.87 in 2022 and *R*^*2*^ = 0.89 in 2023) indicating the robustness of the additive component in QTL interactions. Still, most of the points are located below the observed = expected diagonal suggesting that less-than-additive is a relevant pattern, in particular for combinations of large-effect QTLs. General less-than-additive epistasis is also confirmed by the slopes of regression lines for the expected versus observed of the QTL-pairs, which are significantly lower than 1.00 (0.72 and 0.74 in 2022 and 2023, respectively. Figure 6i, j).

As part of the detailed analyses, we also identified favorable, TSS-increasing QTL alleles, contributed by two of the non-sweet parents in our mapping populations. Both are population-specific (Fig. 5a, Supplementary Table S3). *qTSS1.1* was mapped in the TAD × QME population; this QTL explains 19% of the variation and the QME allele was associated with a 30% increase in TSS (Fig. 6g). *qTSS4.1* was uniquely mapped in the TAD × PI164323 cross. The favorable allele in this QTL is contributed by the non-sweet parent, PI164323, and explained 9% of the phenotypic variation in this population (Fig. 6h).

### Correlation of TSS with ripening behavior and fruit size

An advantage of this broad data set, that we composed from phenotypic data collected from multiple populations and over multiple experiments, is its ability to allow general conclusions to be drawn regarding the correlations of other traits with TSS variation in melon. The list of populations, experiments and traits collected across this study is summarized in Table 1 (and Supplementary Table S1): more than 30,000 experimental units (plants) are included and associated with one or more phenotypic traits. We used ~ 23,000 TSS and average fruit weight (AFW) related data-points that were collected for the same fruits across 12 experiments representing six different populations (Table 1, Supplementary Table S1), to observe that AFW variation is weakly correlated with TSS, explaining on average only 2% of the variation (Table 2). However, we did observe a difference in this response among populations. Interestingly, our data suggest that the correlations are mainly the result of genetic linkage rather than phenotypic or physiological correlations. This is reflected by the fact that in crosses where the sweet parent is larger than the non-sweet, the correlation is positive (e.g. TAD × QME F_4_ and F_6_ (*r* = 0.22), TAD × DUL RIL population (*r* = 0.21)). Whereas, we found weak negative AFW-TSS correlations in the SAS × DOYA F_5_ tails (*r* = − 0.18) and TAD × PI164323 F_4_ population (*r* = − 0.18), where the sweet parents are also potential donors of smaller fruit alleles (Table 2). There is also a notion that length of the development and ripening period is positively correlated with sweetness, such that earliness would be negatively correlated with TSS. This is partly confirmed across more than 19,000 data-points in three populations and seven experiments, where TSS and days to harvest (DTH) data were collected in parallel. In both diversity panels, the half-diallel population and the segregating RIL population, we found significant positive correlations between TSS and days-to-maturity (average *r* = 0.276, Table 2). Another support for a potential moderate correlation between ripening behavior and TSS is through the negative correlation between ethylene emission in ripe fruits (as a measure of climacteric ripening) and TSS in the TAD × DUL RIL population (*n* =  ~ 1,000 fruits, average *r* = − 0.15, Table 2).

**Table 2:** Relations between fruit size or earliness and TSS variation, across multiple populations

Population	Year	*X*	*Y*	Correlation	*P*	*R*^2^	No. Genotypes	No. plants	*r* means
TAD × DUL RILs	2016	DTH	TSS	0.108	0.17192	1%	160	2400	0.20
TAD × DUL RILs	2017	DTH	TSS	**0.226**	**0.00449**	5%	160	2400	
TAD × DUL RILs	2018	DTH	TSS	**0.268**	**0.00116**	7%	160	2400	
*Melo180*	2018	DTH	TSS	**0.390**	**0.00000**	15%	180	2700	0.39
HDA20	2018	DTH	TSS	0.128	0.06518	2%	210	3150	0.31
HDA20	2019	DTH	TSS	**0.380**	**0.00000**	14%	210	3150	
HDA20	2020	DTH	TSS	**0.434**	**0.00000**	19%	210	3150	
TAD × DUL RILs	2016	AFW	TSS	**0.238**	**0.00228**	6%	160	2400	0.21
TAD × DUL RILs	2017	AFW	TSS	**0.179**	**0.02358**	3%	160	2400	
*Melo180*	2022	AFW	TSS	0.064	0.39780	0%	180	2700	0.06
*HDA20*	2018	AFW	TSS	0.003	0.96603	0%	210	3150	0.02
*HDA20*	2019	AFW	TSS	0.006	0.93128	0%	210	3150	
*HDA20*	2020	AFW	TSS	0.063	0.36550	0%	210	3150	
TAD × QME F3	2020	AFW	TSS	0.064	0.44281	0%	218	2180	0.17
TAD × QME F4	2020	AFW	TSS	**0.187**	**0.00680**	4%	218	436	
TAD × QME F6_Tails	2022	AFW	TSS	**0.259**	**0.0389**	7%	64	640	
TAD × PI164323 F4	2021	AFW	TSS	**−0.175**	**0.0511**	3%	132	1980	−0.12
TAD × PI164323 F6_Tails	2022	AFW	TSS	−0.071	0.5732	1%	65	650	
SAS × DOYA F5_Tails	2022	AFW	TSS	−0.180	0.1676	3%	60	600	−0.18
TAD × DUL RILs	2016	ETH	TSS	**−0.168**	**0.03682**	3%	169	2400	−0.15
TAD × DUL RILs	2017	ETH	TSS	−0.129	0.10536	2%	169	2400	

TSS = Total soluble solids, DTH = Days to harvest, AFW = Average fruit weight, ETH = Ethylene emission

## Discussion

### Heritability and mode of inheritance of melon sweetness

Melon sweetness is a complex trait displaying quantitative continuous variation. In addition to genetics, multiple external factors, such as temperature, insolation, soil-type, cultural practices, and fertilization and irrigation regimes interplay and create significant effects on the final sugar content of the mature fruits (Bouwkamp et al. 1978; Leskovar et al. 2001; Huang et al. 2012; Yue et al. 2023). Accordingly, broad-sense heritability of melon sugars and TSS is variable, and significant environment (E) and genotype by environment (G × E) effects are not uncommon in statistical analyses of melon sweetness variation across years and experiments (Argyris et al. 2017; Andrade et al. 2019, 2021). We observed that sugar levels were lower for sweet-fleshed accessions in the field experiment of 2023 than in 2022 (Supplementary Fig. S3). We also observed that although the QTL effects in 2023 were generally lower (Supplementary Table S3), the correlations between years on an accession-mean basis was high (Supplementary Fig. S9), indicative of a moderate to high heritability of TSS in these experiments.

Characterization of mode of inheritance (MOI) of horticulturally relevant traits, such as fruit sweetness, is fundamental in crops where commercial varieties are F_1_ hybrids, as in melon. We performed broad characterization of MOI by analyzing a half-diallel population composed of 190 F_1_s derived from intercrossing of 20 diverse melon inbreds. Using robust TSS data collected over 3 years (Supplementary Fig. S10), we showed that the additive inheritance component is prominent for TSS, such that the parental mean explained 73% of TSS variation in F_1_ hybrids across the 190 hybrid groups (Fig. 2b). Nevertheless, our data confirmed that dominance effect is still significant in many hybrids towards both high- or low-TSS parents (Fig. 2d-h), including a small proportion of hybrids that display over-dominance (|*d/a*|> 1, Fig. 2e). These results are consistent with previous studies that reported on significant general and specific combining ability (GCA and SCA) variance components calculated for sweetness-related traits across different melon panels (Barros et al. 2011; Pouyesh et al. 2017; Akrami and Arzani 2019; Napolitano et al. 2020; Kalb and Davis 2022). From a breeding standpoint, these results imply that pedigree selections for TSS are very effective as long as they are based on replicated measurements taking into consideration the moderate heritability of TSS. Minor dominance-related deviations can still be expected in hybrids.

### Genetic correlations between fruit sweetness and fruit size

In multiple plant species, including cucurbit crops (Levi et al. 2017), human selection during domestication and cultivation improved fruit flavor through various metabolic shifts, including loss of bitterness or acidity and gain of sweetness (Alseekh et al. 2021). Improvement of tomato (*Solanum lycopersicum* L., Solanaceae), however, represents another path, related to the most prominent effect of domestication in this crop: its dramatic increase in fruit size. Interestingly, the strong selection for larger fruit size was associated with lower sweetness of current elite tomato varieties (Tieman et al. 2017) and overall negative correlation between fruit weight and TSS across tomato diversity (Zemach et al. 2023). Tieman et al. confirmed this negative correlation also in an F_2_ population from a cross between a small-fruited, high-flavor line and a large-fruited, modern inbred. The question remains whether this negative relation between fruit size and sweetness in tomato has also an inherent physiological reason or whether it only reflects the outcome of a genetic sweep during long-term selection for larger fruit or linkage in a segregating population. Recently, through targeted knockouts of two protein kinases, Zhang et al. (2024) increased sugar contents of tomato by up to 30% without fruit weight or yield penalty and demonstrated that this relation can be broken. In the current study, we used our dataset to test correlations between sweetness and fruit size (measured as average fruit weight) in melon across multiple populations and experiments. Our results suggest that the correlations are largely genetic rather than phenotypic or physiological correlations between traits, as they can go in both directions based on the fruit size and sweetness profile of parental lines (Table 2), pointing to a genetic sweep as the greater contributor. Variable correlations between TSS and fruit weight were also reported from smaller-scale experiments in other studies in melon (Eduardo et al. 2007; Ramamurthy and Waters 2015; Pouyesh et al. 2017; Pereira et al. 2018).

We also showed that there is a positive correlation between days-to-maturity and TSS and a weak negative correlation between ethylene emission and TSS (Table 2), most likely confirming the dependence of sucrose accumulation during fruit development on the length of the fruit-development window (Burger et al. 2000). The weak correlation of sweetness and ripening behavior in segregating populations is consistent with the fact that there are sweet melons in both climacteric and non-climacteric types. Taken together, these results support the effectiveness of breeding for sweet melons in various fruit sizes or ripening-behavior types.

### The singular and combined effects of melon TSS QTLs

At its core, plant breeding is about stacking favorable allelic combinations to produce desired improved plant phenotypes. While such genetic improvements were achieved over thousands of years through direct phenotypic selections, in the past 30 years it has become possible to genetically dissect traits and introduce breeding improvement by identifying and stacking discrete genetic components using linked molecular markers (Ribaut and Hoisington 1998). Therefore, in addition to identifying the trait loci and estimating their allelic effects, it is critical to obtain estimates for their interactions. Here, in addition to the singular effect of each QTL, we analyzed and present examples of QTL pairs that show complete additivity of their independent effects when combined (non-significant QTL × QTL interactions, Fig. 6a-d). Similarly, the pyramiding of favorable alleles of 3 QTLs resulted in a more than 70% increase in TSS as compared with the triple non-favorable haplotypes, in two populations (Fig. 6e-f).

Finally, we performed a broad analysis of interaction pattern with the 64 QTL-pair combinations, by plotting the expected sum of the effects for each QTL-pair against the observed TSS value (Fig. 6i-j). The results demonstrated the prominent cumulative nature of TSS QTLs, with a significant difference between the allelic effect of a single QTL and QTL pairs (Fig. 6k-l). At the same time, QTL pairs display a robust pattern of less-than-additive epistasis, reflected by the fact that the slope of the regression of expected versus observed TSS was < 1 (Fig. 6i-j). Such a diminishing effect of QTL epistasis was also previously described for TSS and yield-related traits in tomato (Eshed and Zamir 1996; Gur and Zamir 2015), and is supported by an earlier view regarding trait canalization as an evolutionary mechanism to keep phenotypes within a conserved range (Waddington 1942). More evidence for the less-than-additive model and cumulative nature of TSS QTLs is in the observation that even through selection of extreme high- and low-TSS tail segregants in our BSA-Seq analyses, we still found balanced proportions of allelic combinations for different QTL-pair haplotypes. This confirms that the high TSS tail segregants are not simple selection of favorable allelic stacks of all major QTLs, but rather reflect varying allelic QTL combinations that produce parallel outcomes.

### Towards a pan-QTLome for melon sweetness

A fundamental theoretical question in quantitative genetics is how many discrete elements (genes) shape the overall phenotypic variation of a quantitative trait within a species. In forward genetics, the common path towards an answer goes through QTL mapping. QTL is a statistical event that describes a significant association of a genomic region with trait variation, identified under a specific population and experimental setup, and as such represents only a snapshot of the overall causative variation. In recent years, reference genomes and even pan-genomes have become the new standard in the research toolbox of crop geneticists (Xie et al. 2024). Additionally, the recent progress in the ability to easily and cost-effectively analyze sequence variation for genotyping of populations is a significant technological leap. However, these advancements are not necessarily paralleled with improved efficiency and resolution of whole-genome QTL mapping, which is still inherently a demanding and costly effort, fully dependent on phenotyping of large populations in replicated experiments. Multi-experiment genome-wide QTL mapping in a specific cross can theoretically describe the full repertoire of loci associated with trait variation between particular parents, namely trait QTLome (Salvi and Tuberosa 2015), which is analogous to genome, transcriptome or metabolome. Accordingly, the comprehensive description of multiple QTLomes for a trait within a species can be termed "Pan-QTLome", and its construction requires extensive characterization of QTLs in multiple diverse crosses that represent a broad spectrum of parental and allelic combinations. Due to the limitations of genome-wide association analysis (GWAS) for QTL mapping (Myles et al. 2009), multi-parental segregating populations such as nested association mapping (NAM) design (Yu et al. 2008), or multi-parent advanced generation inter-cross (MAGIC) (Pascual et al. 2015) are common frameworks designed to facilitate simultaneous QTL analysis in multiple parents through linkage mapping (Scott et al. 2020). However, practically, for vegetable crops of moderate economic importance, such as melon, pan-QTLome data on complex, horticulturally important traits is more likely to be generated over time by the crop community through a decentralized, non-coordinated research effort, in a way that is more similar to a social-network economy concept, rather than through a specific large-scale project or initiative as in model crops, such as flowering time in maize (Buckler et al. 2009). In that respect, the availability of a high-quality reference (pan) genome, as an efficient common anchor for QTLs detected across different populations, is pivotal for construction of a pan-QTLome. Furthermore, this concept emphasizes the importance of detailed and standardized sharing and deposition of genetic and phenotypic data, as well as a complete description of the experimental germplasm (Zamir 2013; Anon 2015) for future construction of a phenotypic and QTL database as a complementary layer to the cucurbit genomics database (CuGenDBv2, http://cucurbitgenomics.org/v2/) (Yu et al. 2023).

In the current study, we integrated QTL mapping results from 5 populations that we analyzed, with 15 additional published melon sweetness QTL studies conducted from 2004 to 2023 by the melon genetics community. Collectively, the 20 populations capture a broad spectrum of crosses and allelic combinations as well as different population structures (Fig. 5b, Supplementary Table S4 and S5), and therefore can potentially represent a large proportion of sweetness-related QTL allelic variation in *Cucumis melo*. While some of the QTL intervals are anchored in our integrated map to large chromosomal segments (Fig. 5a, Supplementary Table S4 and S5; see methods section), it is logical to assume that overlapping QTL intervals represent a common QTL across populations and experiments. Through this analysis, as in core versus dispensable genes in pan-genome analysis, QTLs can be classified as common, rare or unique based on the magnitude of their effect and frequency on the pan-QTLome. Interpretation, though, can be more complicated. For example, a rare QTL such as *qTSS1.2* on chr.1, which was significant only in the TAD × PI164323 F_6_ population (Fig. 3, 5 and Supplementary Table 3), could be a result of such a rare allele, but at the same time the result can be explained as an effect of a modifier gene sensitive to epistatic interactions.

In the future, with more sweetness-related QTL studies aligned to the melon genome as presented here, a pan-QTL analysis that corresponds with cumulative graphs of core and pan genes commonly presented in pan-genome analyses (Liu et al. 2020; Wang et al. 2023) can be a potential way to get an informed estimate of the actual number of QTLs (genes) participating in shaping the natural variation of fruit sweetness in *C. melo*.

Our integrated QTL analysis also highlighted the importance of *qTSS3.1*, which appears to be common in 11 populations, and *qTSS8.4*, which is present in 7 populations (Fig. 5a, d and Supplementary Table S5). Noteworthy is the common QTL interval reported in previous studies on chromosomes 10 (reported in 7 experiments) that was not mapped in any of the populations in the current study (Fig. 5a and Supplementary Table S5). This suggests that the causative allelic variation in this QTL was not segregating in our populations. These results further emphasize the importance of analyzing multiple populations to capture maximal proportion of QTLs contributing to sweetness variation.

### Conclusions and breeding implications

The integrated investigation presented here, that combines comprehensive analysis of mode-of-inheritance of TSS, characterization of correlations of TSS with other morphological and physiological fruit traits, and broad comparative analysis of sweetness-related QTLs, provides a useful tutorial for melon breeders as they approach further into stacking of favorable sweetness alleles using marker-assisted selection. Organization of the current knowledge on the genetic architecture of melon sweetness in a unified framework and the understanding that a manageable number of major-effect QTLs are explaining most of the sweetness variation, is consistent with the practical 'breeders experience' of strong response to selection for this trait. Definition of the major sweetness-related loci in *Cucumis melo* can also be useful for geneticists in prioritization of target genomic regions for future isolation of causative genes that can then be followed by effective characterization of allelic variation towards development of an optimized QTL stacking strategy.

## Supplementary Information

Below is the link to the electronic supplementary material.

Supplementary file1 (PDF 2106 KB)

Supplementary file2 (XLSX 45 KB)

## Data Availability

The data supporting the findings of this study are available within the paper and within its supplementary materials published online. Supplementary Datasets are available online at http://datadryad.org/stash/share/kdaVAkTVER-WMqOpb_nnUcM0tZXz8cTOySYl1FhGnfI
