# Supplementary material for: Meta genetic analysis of melon sweetness
Source: Theor Appl Genet. 2025 Mar 11;138(4):68. doi: 10.1007/s00122-025-04863-6 (PMC11897113; doi:10.1007/s00122-025-04863-6)
Supplement: Supplementary file 1 — Supplementary file1 (PDF 2106 KB) [file 122_2025_4863_MOESM1_ESM.pdf]

Supplementary Figure S1: Six melon inbred lines used as parents of populations 3-6

Sweet accessions

Non-sweet accessions

Tam Dew (TAD)

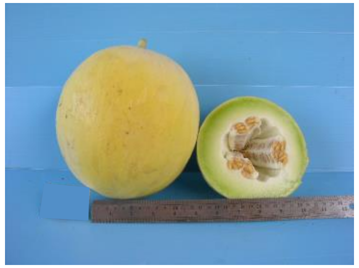

PI 164323

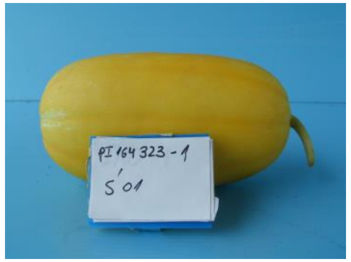

Sakata's Sweet (SAS)

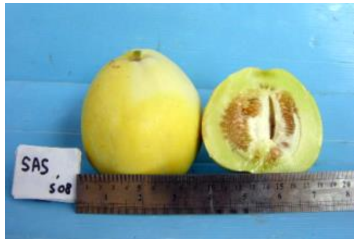

Qishu Meshullash (QME)

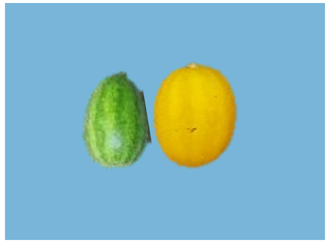

Dulce (DUL)

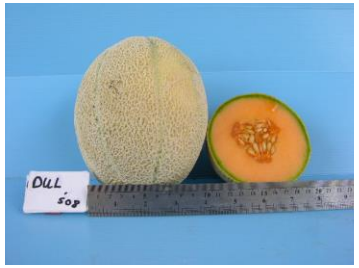

Doya's Faqqous (DOYA)

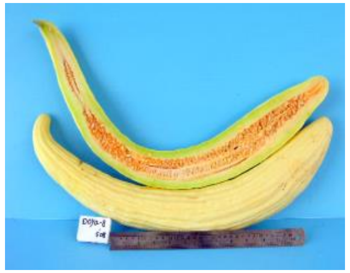

Subsp.	Group	Abbreviation	Accession	Sweetness	Population #3	Population #4	Population #5	Population #6
melo	Inodorous	TAD	Tam Dew	Sweet	X	X	X	
melo	Reticulatus	DUL	Dulce	Sweet	X			
collosus	[feral]	QME	Qishu Meshullash	Non-sweet		X		
melo	Adzhur	PI 164323	PI 164323	Non-sweet			X	
agrestis	Makuwa	SAS	Sakata's Sweet	Sweet				X
melo	Flexuosus	DOYA	Doya's Faqqous	Non-sweet				X

Supplementary Figure S2: Sampling fruit-flesh cylinders for TSS measurements in the current study.

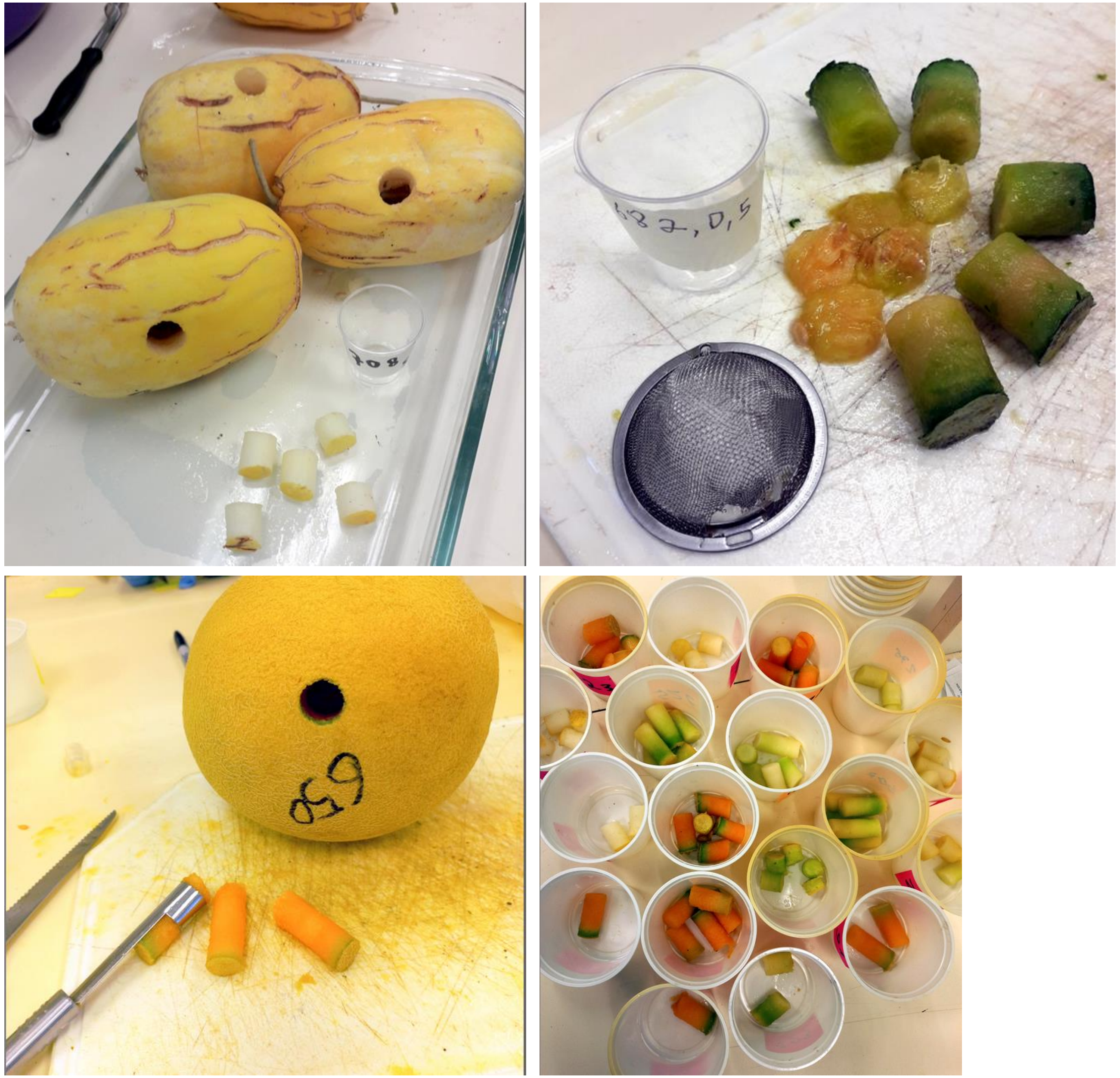

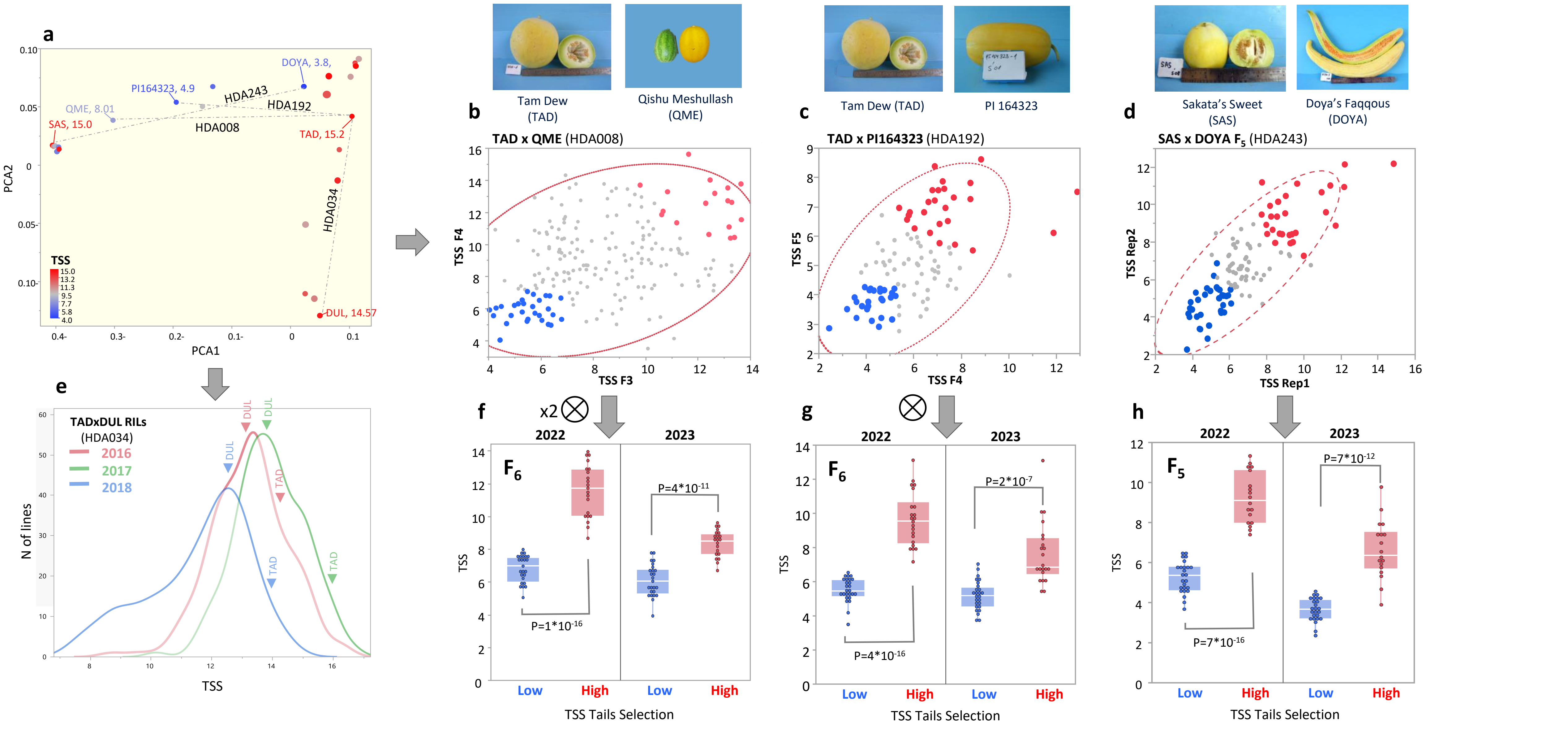

Supplementary Figure S3: Four bi-parental populations developed and analyzed for BSA-Seq and GBS-based QTL mapping. (**a**) TSS projected on the genetic PCA plot of the 25 diverse core accessions. Dashed lines connect between parents of segregating populations developed and used in this study. TSS values are shown next to the accession abbreviations. (**b**) Fruits of parental accessions and correlation between F_3 and F_4 TSS in the TAD × QME population. Blue points are the selected low-TSS lines. Red points are the selected high-TSS lines. (**c**) Fruits of parental accessions and correlation between F_4 and F_5 TSS in the TAD × PI 164323 population. Blue points are the selected low-TSS lines. Red points are the selected high-TSS lines. (**d**) Fruits of parental accessions and correlation of TSS between replications at the F_5 in the SAS × DOYA population. Blue points are the selected low-TSS lines. Red points are the selected high-TSS lines. (**e**) Frequency distributions of TSS in the TAD × DUL RIL population in three field experiments. Parental TSS in each experiment is indicated. (**f**) Validation of differential TSS of selected tails in F_6 lines of the TAD × QME population in the 2022 and 2023 replicated field experiments. Each point is the average TSS of ~10-15 fruits. (**g**) Validation of differential TSS of selected tails in F_6 lines of the TAD × PI 164323 population in the 2022 and 2023 replicated field experiments. Each point is the average TSS of ~10-15 fruits. (**h**) Validation of differential TSS of selected tails in F_6 lines of the SAS × DOYA population in the 2022 and 2023 replicated field experiments. Each point is the average TSS of ~10-15 fruits.

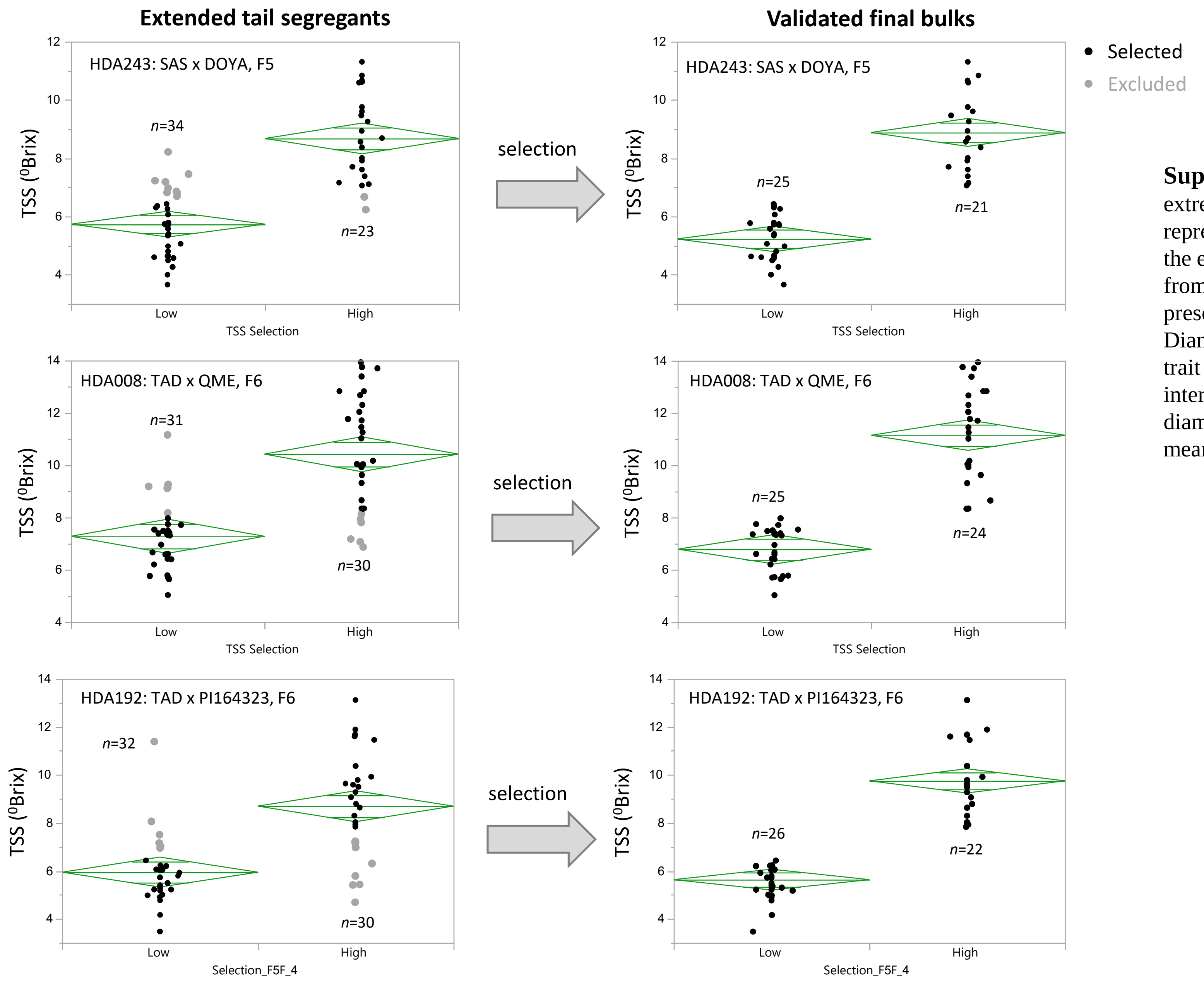

Supplementary Figure S4: TSS tail-segregant validations and selection of extreme lines for bulks, for QTL-Seq analysis in three populations. Each dot represents the mean TSS value from 10 fruits of an F_5 or F_6 line. On the left are the extended tails selected for validation. Gray dots are lines that were excluded from the final set to ensure non-overlapping bulks for QTL-Seq analysis, as presented on the right. The number of lines in each group is shown (*n*). Diamonds show the trait mean and confidence interval. Mid-horizontal line is the trait mean. The top and bottom of each diamond span the 95% confidence interval for the mean of each group. The lines near the top and bottom of the diamonds are overlap marks for visual statistical difference between group means.

a) Convincing InDel

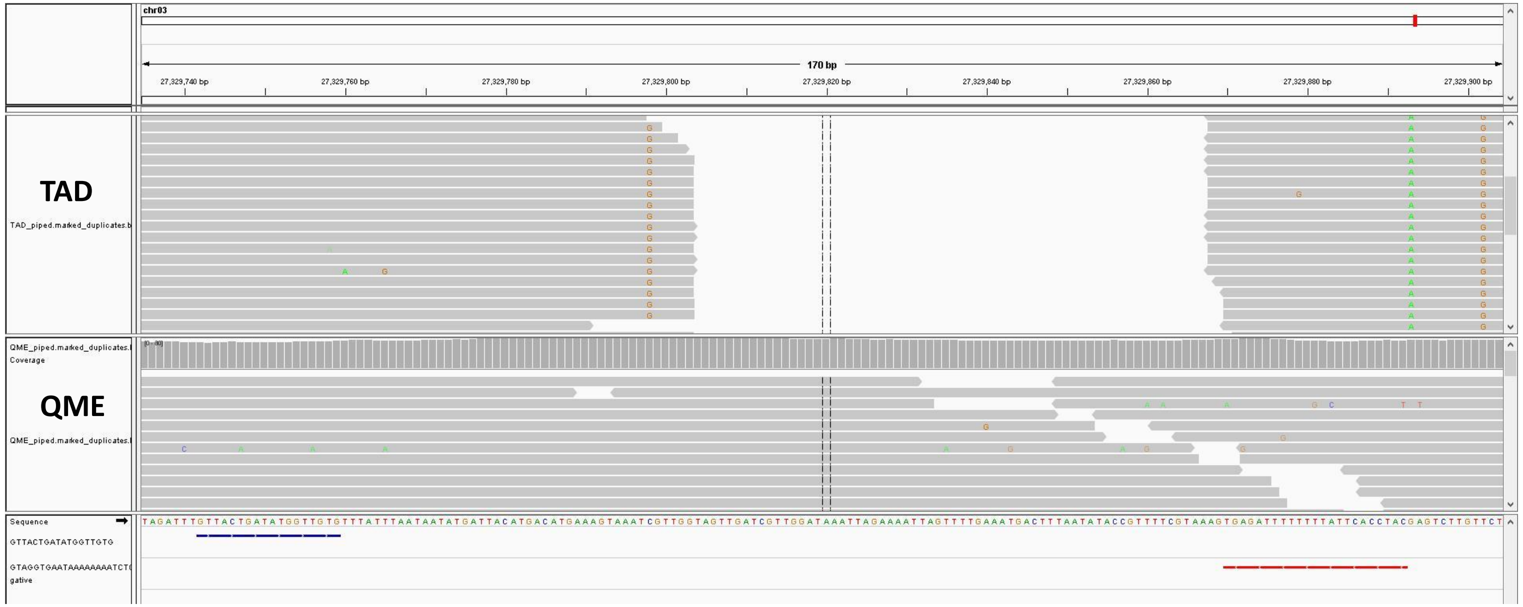

b) Ambiguous InDel

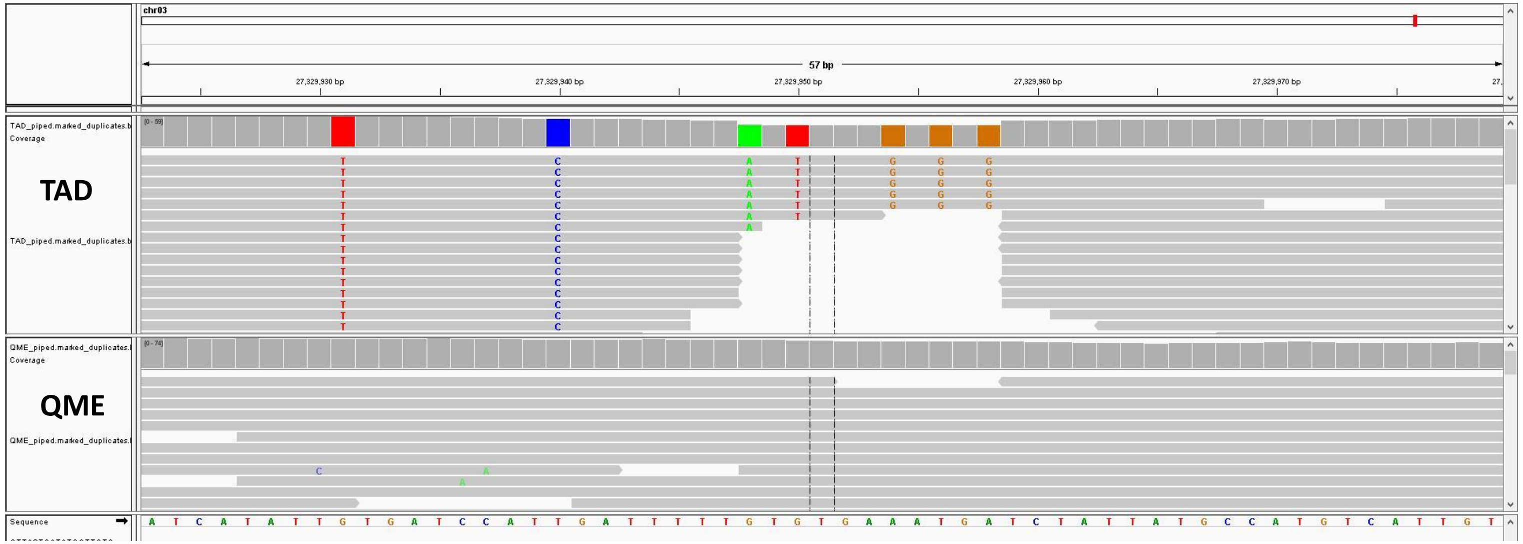

Supplementary Figure S5: Review of raw sequence variation in putative InDels identified for QTL validation, based on the database and pipeline described by Oren et al. (2022). **a**) . Integrated Genomic Viewer (IGV) screenshot of clear InDel signature between the parental accessions TAD and QME. **b**) Ambigous InDel detected by the algorithm but received low priority for PCR marker development. Gray horizontal bars represent Illumina reads.

Supplementary Fig. S6: Total sugar variation is mostly explained by variation in sucrose in the *Melo180* diverse collection. **a**) Correlation between sucrose and total sugars across 177 diverse accessions. Each dot is the mean of 10 - 15 fruit samples collected from 3 different plots. Red ellipse indicates the bivariate normal density at 95% coverage **b**) Correlation between glucose and total sugars. **c**) Correlation between fructose and total sugars.

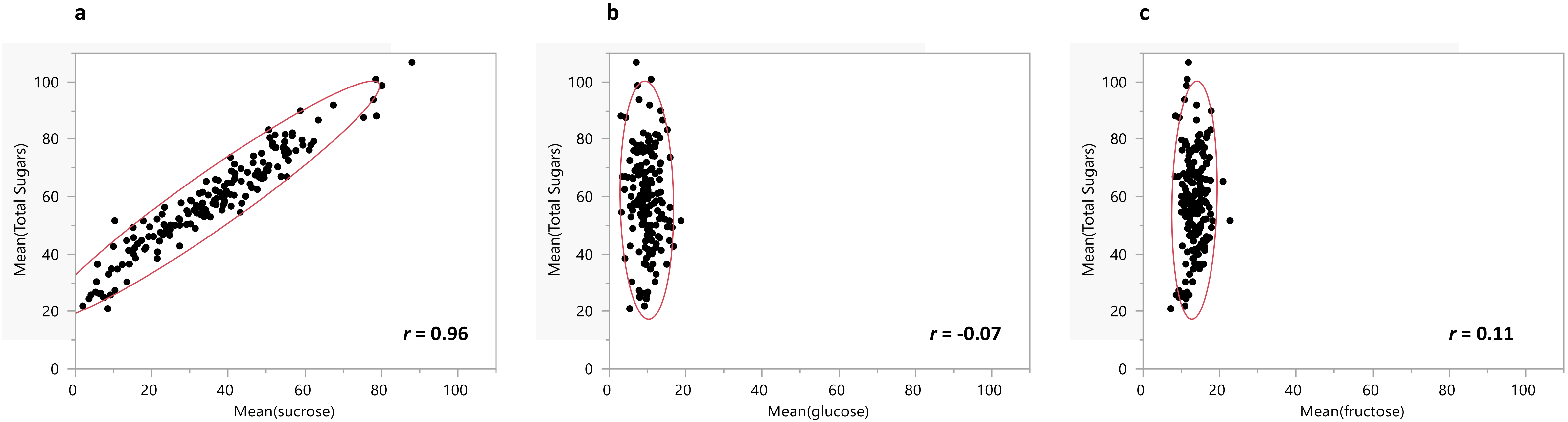

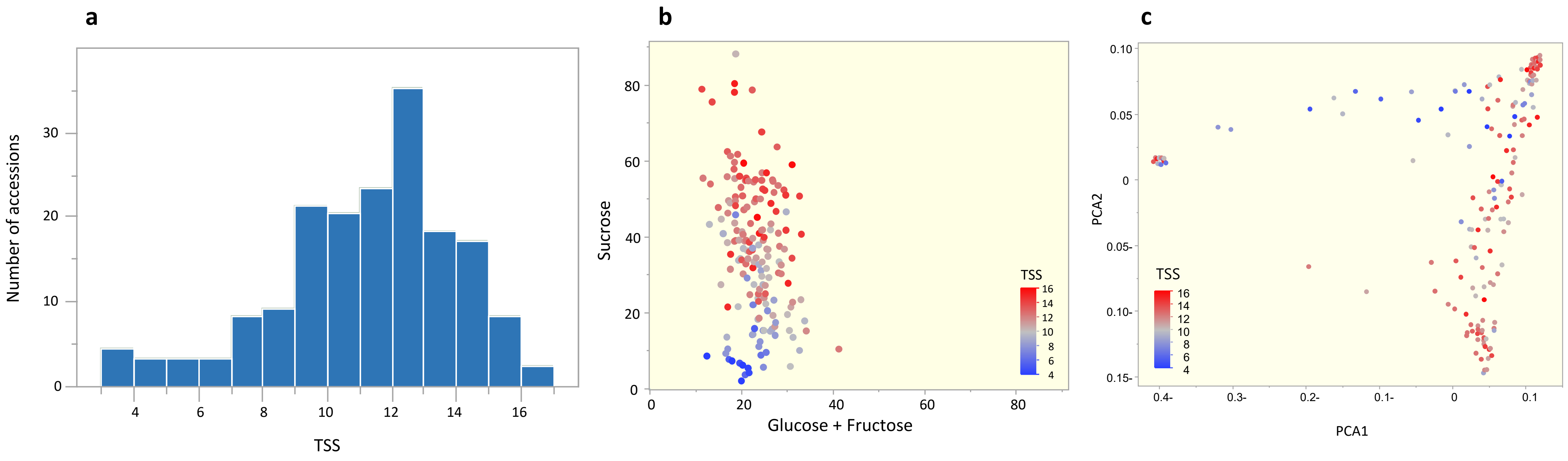

Supplementary Fig. S7: Characterization of TSS and sugar composition in the *Melo180* diverse collection. (**a**) Frequency distribution of TSS in mature fruits of 177 diverse accessions. (**b**) Correlation between total monosaccharides (glucose and fructose) and sucrose across 177 diverse accessions. Each dot is the mean of 10 - 15 fruit samples collected from 3 different plots. The color gradient reflects TSS values. (**c**) TSS projected on the genetic PCA plot of the 177 diverse accessions.

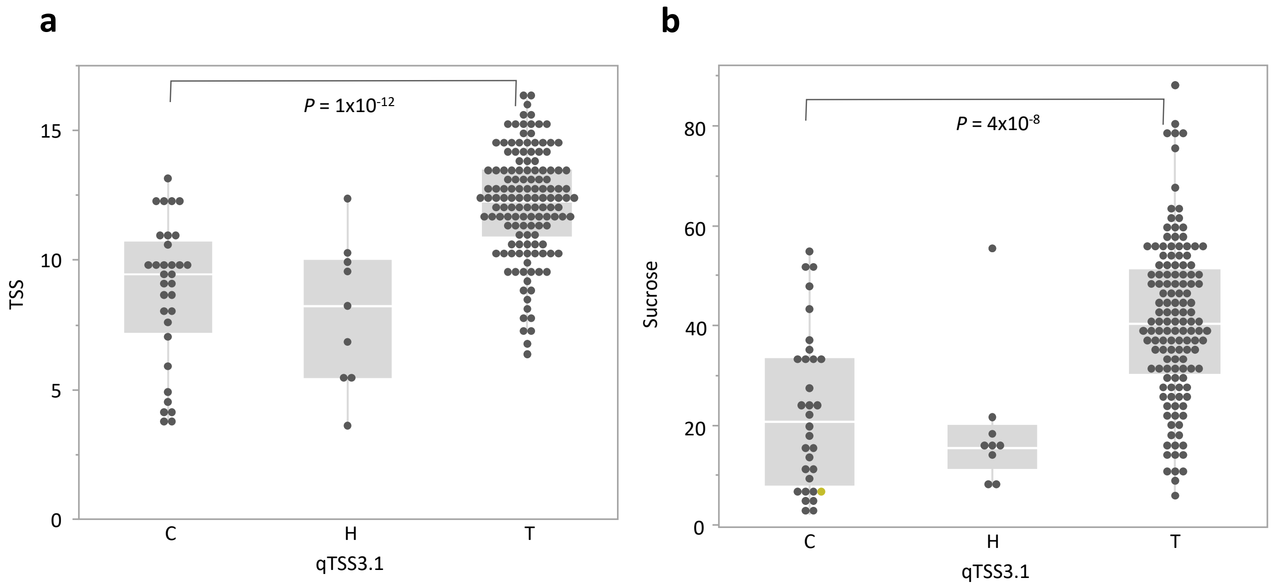

Supplementary Fig. S8: Allelic effect of qTSS3.1 in the *Melo180* GWAS panel. (**a**) Genotypic comparison plot for the association of the SNP at the peak of the *qTSS3.1* QTL with TSS across 177 diverse accessions. Each point represents an accession mean. The *P* value is for the TSS comparison between homozygote genotypes. (**b**) Genotypic comparison plot for the association of the SNP at the peak of the *qTSS3.1* QTL with sucrose content. Each point represents an accession mean. The *P* value is for the sucrose concentration comparison between homozygote genotypes.

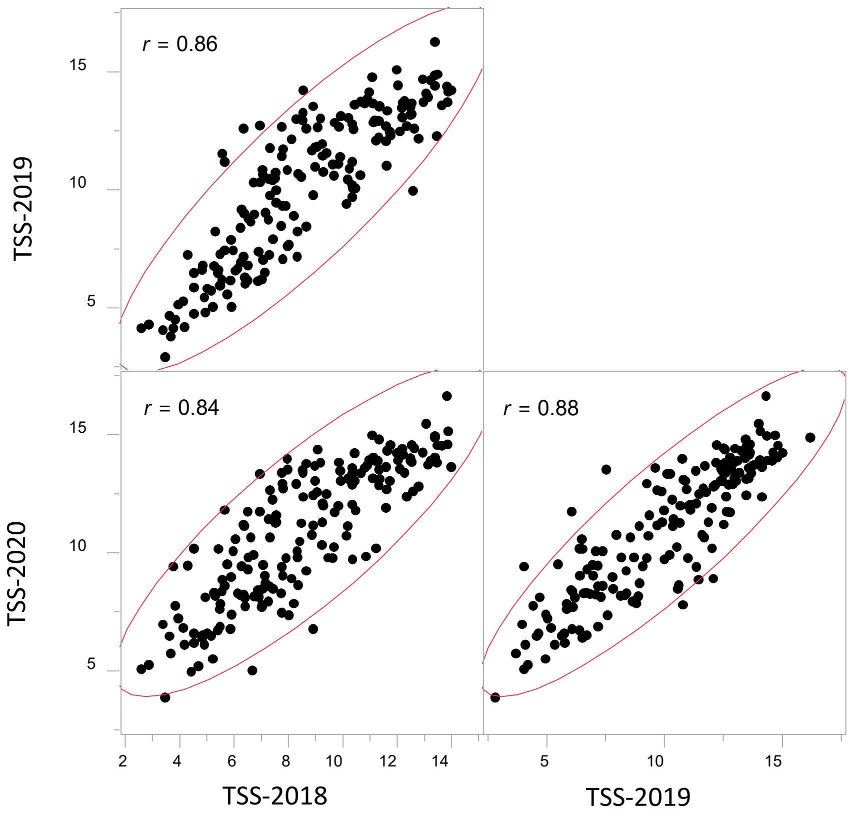

Supplementary Fig. S9: Correlations of TSS between years in the *HDA20* population. Each dot is the mean of 10-15 fruit samples collected from 3 different plots. Red ellipse indicates the bivariate normal density at 95% coverage.

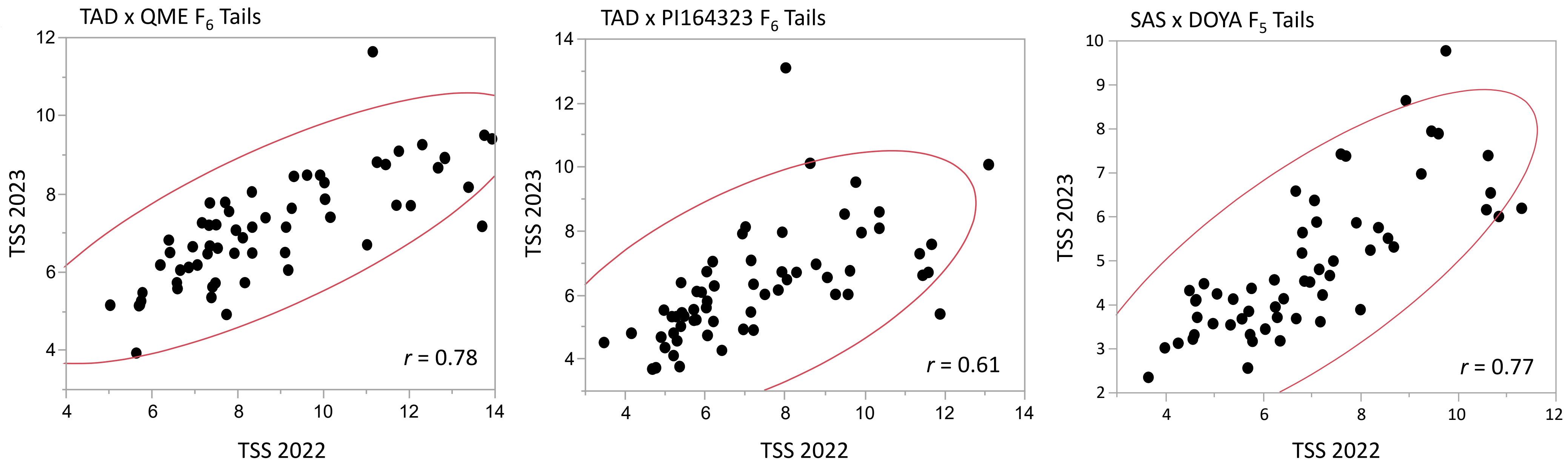

Supplementary Fig. S10: Correlations of TSS between the 2022 and 2023 field trials on tail-segregants in 3 F_5 and F_6 populations. Each dot is the mean of 10 fruit samples collected from 2 different plots. Red ellipse indicates the bivariate normal density at 95% coverage.
